# Annotation and functional clustering of circRNA expression in rhesus macaque brain during aging

**DOI:** 10.1038/s41421-018-0050-1

**Published:** 2018-09-18

**Authors:** Kaiyu Xu, Dong Chen, Zhengbo Wang, Jian Ma, Jian Zhou, Nanhui Chen, Longbao Lv, Yongtang Zheng, Xintian Hu, Yi Zhang, Jiali Li

**Affiliations:** 1 Key Laboratory of Animal Models and Human Disease Mechanisms of Chinese Academy of Sciences & Yunnan Province, Kunming Institute of Zoology, the Chinese Academy of Sciences, Kunming, Yunnan 650223 China; 2 Kunming College of Life Science, University of Chinese Academy of Sciences, Kunming, 650223 China; 3 Center for Genome Analysis, ABLife Inc., Wuhan, Hubei 430075 China; 4 CAS Center for Excellence in Animal Evolution and Genetics, Chinese Academy of Sciences, Kunming, Yunnan 650223 China; 5 Laboratory for Genome Regulation and Human Health, ABLife Inc., Wuhan, Hubei 430075 China; 6 Kunming Primate Research Center of the Chinese Academy of Sciences, Kunming, Yunnan 650223 China

## Abstract

The abundance and function of circular RNAs (circRNAs) in mammalian brain have been reported, but their alterations in the biology of brain aging remain elusive. Here, using deep RNA profiling with linear RNA digestion by RNase R we explored a comprehensive map of changes in circRNA expression in rhesus macaque (*macaca mulatta*) brain in two age groups from adult (10 y) to aged (20 y) periods. Total 17,050 well expressed, stable circRNAs were identified. Cluster analysis reveals that dynamic changes in circRNA expression show the spatial-, sex- and age-biased specificities. On the basis of separate profiling of the RNAs, age-related circRNAs show differential correlation to host mRNA expression. Furthermore, two voltage-dependent L- and R-type calcium channel gene-derived circCACNA2D1 and circCACNA1E negatively regulate their host mRNA expression. Our results demonstrate the power of changes in circRNA expression to reveal insights into a potentially circRNA-mediated regulatory mechanism underlying the biology of brain aging.

## Introduction

Aging leads to changes in brain function as well as many other physiological activities^1^. Brain aging is characterized by changes in the neuronal physiology, from molecular, to cell and functional levels^2^. One of the biggest obstacles in the study of brain aging is there are no good markers which classify normal versus pathological brain aging^3,4^. Recent studies demonstrate that local RNA including mRNA-, miRNA- and lncRNA-mediated regulation is heavily involved in synaptic development and function^5–9^.

Circular RNA (circRNA) is formed by head-to-tail splicing with the covalent joining the 5′ end of one exon with the 3′ end of another^10–16^. It has recently emerged as a novel class of transcripts with complex tissue- and spatiotemporal expression patterns^17–20^. With deep sequencing of ribosomal RNA (rRNA)-depleted RNA, in combination with other biological and computational tools, this has led to the identification of thousands of new circRNAs in organisms ranging from Archaea to human^21–24^. Alternatively, linear RNA digestion by the RNA exonuclease RNase R method was also employed to identify and enrich circRNAs^20,25^.

The phenomenon in which miRNA ‘sponges’ sequestering miRNAs and prevents their interactions with target mRNAs was contributed by a couple of circRNAs^18,23^. The best known ones so far include a particular type of circRNA namely “circular RNA sponge for miR-7” (ciRS-7/CDR1as) which is the inhibitor of miR-7 microRNA—known to regulate various diseases like, cancer, neurodegenerative diseases, diabetes, and atherosclerosis^26^. Similarly, another circRNA molecule called circMbl modulates the ratio of linear mRNA by competing with linear muscleblind gene through which it is synthesized^13^. In addition, circRNAs could also sequester RNA-binding proteins (RBPs) and thereby regulate the intracellular transport of associated RBPs or RNAs^27^. Recently, two studies suggest that circRNAs could be translated in a cap-independent way, as demonstrated both in vitro and in vivo^28,29^.

CircRNAs are highly enriched in the mammalian brain^30^. Recent studies indicate that circRNAs might service as a new regulatory network that may contributes to brain development, maturity, and aging^30–32^. It is reported that a subset of circRNAs in monkey muscle were significantly repressed as muscle aging^33^. Loss of circRNA CDR1as locus in mouse causes miRNA deregulation and affects brain function^26^. Thus, circRNAs represent a heterogeneous and dynamic class of noncoding transcripts that likely regulate brain function via as yet undiscovered and diverse mechanisms. However, the expression pattern and function of the majority of circRNAs in the brain, especially in primates, remains unknown. Indeed, it is a huge task to explore whether alterations in circRNA expression and function are involved in the process of brain aging.

Aging probably derives from a relationship between environmental factors, mutations, and regulation of gene expression. While in vitro and in vivo studies on mammals allow hypotheses that may be useful for the understanding of aging events^34^, there is no theory for explaining the biological age-related and age-dependent changes in human brain. Nonhuman primates have served as one of the most valuable models for modeling human diseases and developing therapeutic strategies due to their close similarities to human in genetic and physiological features^35^.

In this study, to systematically identify and analyze dynamic changes in circRNA expression in rhesus macaque brain during aging, we sequenced linear RNAs–depleted RNAs from multiple brain compartments in the 10 and 20 years old rhesus macaque brain smaples, which indicated the states of brain aging, and performed subsequent computational analyses and experimental validation. Our findings reveal that circRNAs are highly abundant in rhesus macaque brain, and changes in circRNA expression show spatiotemporal- and sex-biased specificities during aging, and age-related circRNA correlate with their host mRNA expression.

## Results

### Genome-wide identification of circRNAs in rhesus macaque brain

**Fig. 1:**
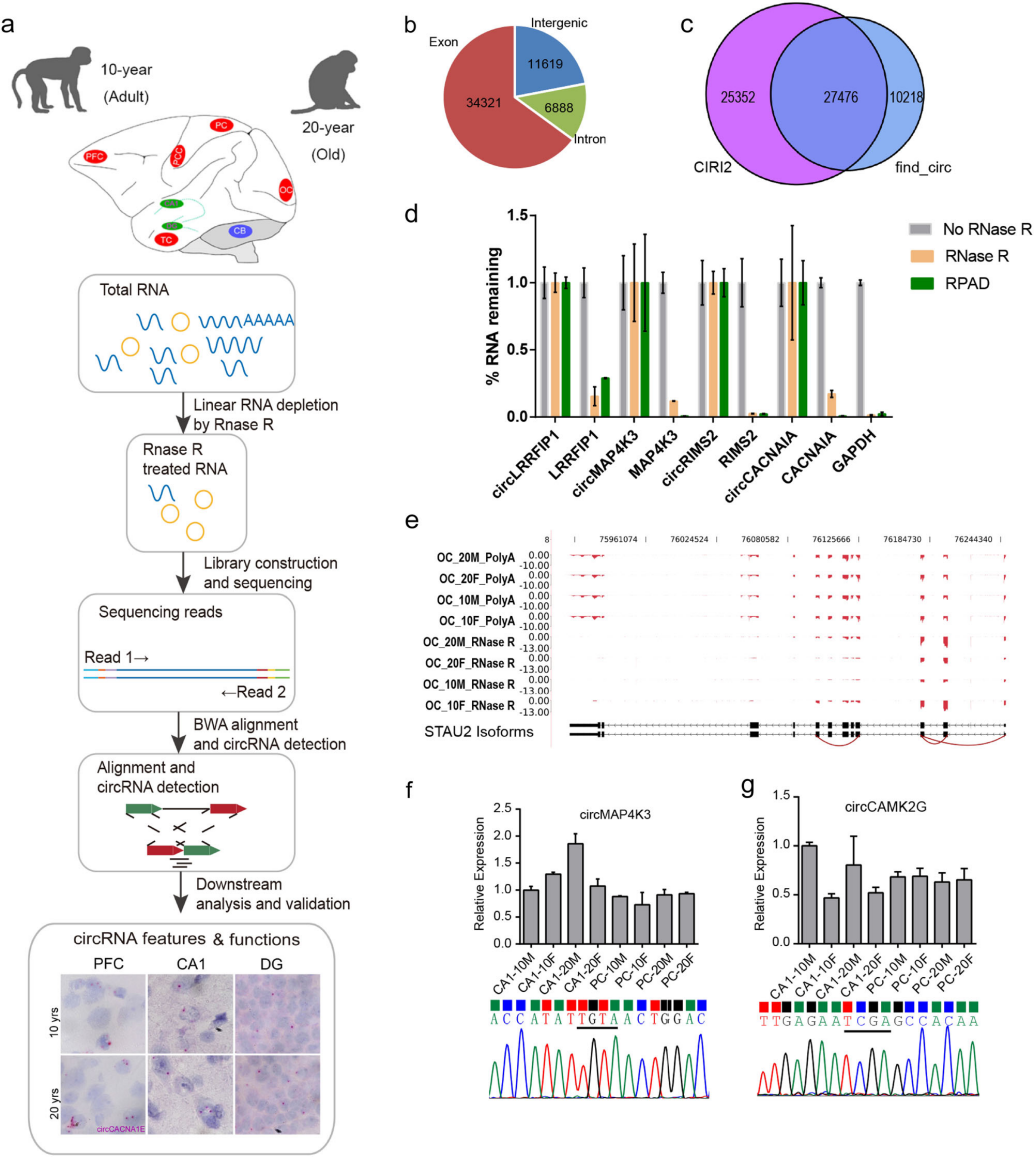
**Profiling of circRNA expression in rhesus macaque brain**. **a** Pipeline of library construction, data analysis and functional exploration for macaque brain-expressed circRNAs in this study. **b** Pie chart showing the genomic distribution of all predicted circRNAs from eight macaque brain regions. **c** Venn diagram showing the overlapping distribution between CIRI2 and find_circ predicted circRNAs. **d** Subsets of circRNAs and mRNAs were quantifed by RT-qPCR analysis with (brown) or without (gray) RNase R treatment, as well as following RPAD (green) method. *GAPDH* was shown with only linear mRNAs. **e** Reads distribution of circSTAU2 from OC region showing the efficient linear mRNA digestion by RNase R. Back splice sites were linked together by red curve. The top four tracks of PolyA selected RNA-seq data from Liu et al.^40^ were shown as background. The *Y* axis is the normalized depth for mapping result. **f**, **g** RT-qPCR (top panel) and Sanger sequencing (bottom panel) validated the presence of two circRNAs specially detected by CIRI2 with the correlated samples for sequencing. Linear RNA of *GAPDH* was used as internal reference, and relative value of each sample was normalized by the first sample in each panel. The bases above the black line represent the back-splicing site

To systematically determine the pattern of circRNA expression in the rhesus macaque brain during aging, we deep-sequenced RNA samples with linear RNAs depleted from eight anatomical brain compartments including prefrontal cortex (PFC), posterior cingulate cortex (PCC), temporal cortex (TC), parietal cortex (PC) and occipital cortex (OC), hippocampus (CA1), and dentate gyrus (DG), and cerebellar cortex (CB) of rhesus macaque across the two ages (10 y and 20 y) (Supplementary Table S1). Research pipeline of our study was briefly concluded in Fig. 1a. Candidate circRNAs with head-to-tail junctions were detected by the CIRI2 software^36^ with the threshold of two or more head-to-tail junction reads in each sample. The DESeq normalization method^37^ was used to quantify circular transcript expression level. In total, we detected 52,828 distinct circRNA candidates spliced from 8147 genes as well as intergenic regions from all macaque brain samples. Brain-expressed circRNAs were usually derived from annotated exons (34,321, 64.97%). Due to the incomplete annotation of macaque, circRNAs derived from the intergenic region (11,619, 21.99%) still occupied reasonable quantity (Fig. 1b). Next, we further utilized find_circ (version 1.2)^23^ to re-predict circRNAs for our data. 37694 circRNAs were detected by find_circ, including 30684 circRNAs (81.40%) with two or more head-to-tail junction reads. Although more than 80% circRNAs by find_circ were re-detected by CIRI2, about half of the circRNAs (48.0%) detected by CIRI2 were not included in the find_circ set (Fig. 1c).

It has been suggested by some studies that RNase R treatment may not be sufficient enough to remove the linear RNA, which could consequently resulted in inaccurately quantitative analysis of circRNAs^38,39^. To assess the efficiency of RNase R treatment in this study, we tested the residual linear RNAs in our samples by using RNase R alone or a recently published RPAD method^39^. It is obvious that linear mRNAs were effectively digested by both RNase R and RPAD methods, when compared to the respective circRNAs. RPAD was more efficient than RNase R alone for two of the four tested linear mRNAs (Fig. 1d). Generally, less than 20% of mRNAs were left after RNase R treatment alone, implying the high efficiency of RNase R treatment. To further illustrate the linear RNA digestion efficiency and the confidence of the detected cirRNA, we presented the reads distribution map of both circRNA-seq reads and RNA-seq reads from polyA-puridied mRNAs for one circRNA host gene, *STAU2*. Exon regions of circRNA origination were with abundant reads, while exon regions out of circRNA origination were with little transcriptional signal (Fig. 1e). As a control, the polyA-enriched RNA-seq data of the same samples from our previous study^40^ showed abundant transcriptional signal in non-circRNA regions (Fig. 1e), illustrating the efficient linear RNA digestion by RNase R.

Splicing sites of circRNAs typically enclose three exons. While the median number of circRNA isoforms per gene in macaque brain samples is five, 1212 genes give rise to ten or more circular isoforms. Similar to the previous study^30^, the majority of head-to-tail junctions were supported by few reads (two to ten), we also detected 7504 (14.20%) circRNAs supported by more than 100 junction-spanning reads (Supplementary Fig. S1a). To confirm the data reliability of circRNA prediction, we downloaded and reanalyzed the mouse data from the previous study^30^ with CIRI2. Over 80% circRNAs detected by Rybak et al. were also detected by CIRI2 (Supplementary Fig. S1b), illustrating the sensitivity of CIRI2. While the 3093 circRNAs detected specifically by CIRI2 also implied the higher discovery efficiency but also the potential false discovery rate. Meanwhile, the overlapped circRNAs exhibited higher abundance than the non-overlapped circRNAs (Supplementary Fig. S1c), which implied that circRNA with the higher expression level could be more easily detected. The higher number of rhesus macaque brain circRNA candidates may partially be explained by the higher sequencing depth.

To independently test the confidence of the CIRI2-detected circRNAs in this study, we randomly selected 24 circRNAs for validation by RT-qPCR with divergent primers, including eight circRNAs only detected by CIRI2 and 16 circRNAs by both methods. All of these circRNAs were validated by RT-qPCR (Supplementary Table S3). Then eleven randomly selected circRNAs from the above 24 circRNAs were subjected to Sanger sequencing (Supplementary Fig. S1d), all showing back-spliced junction sequences consistent with the prediction, including three circRNAs only identified by CIRI2 (Fig. 1f, and Supplementary Fig. S1e), and eight circRNAs detected by both methods (Supplementary Fig. S1f-k).

### Dynamic expression pattern of circRNAs and high conservation between human and macaque

**Fig. 2:**
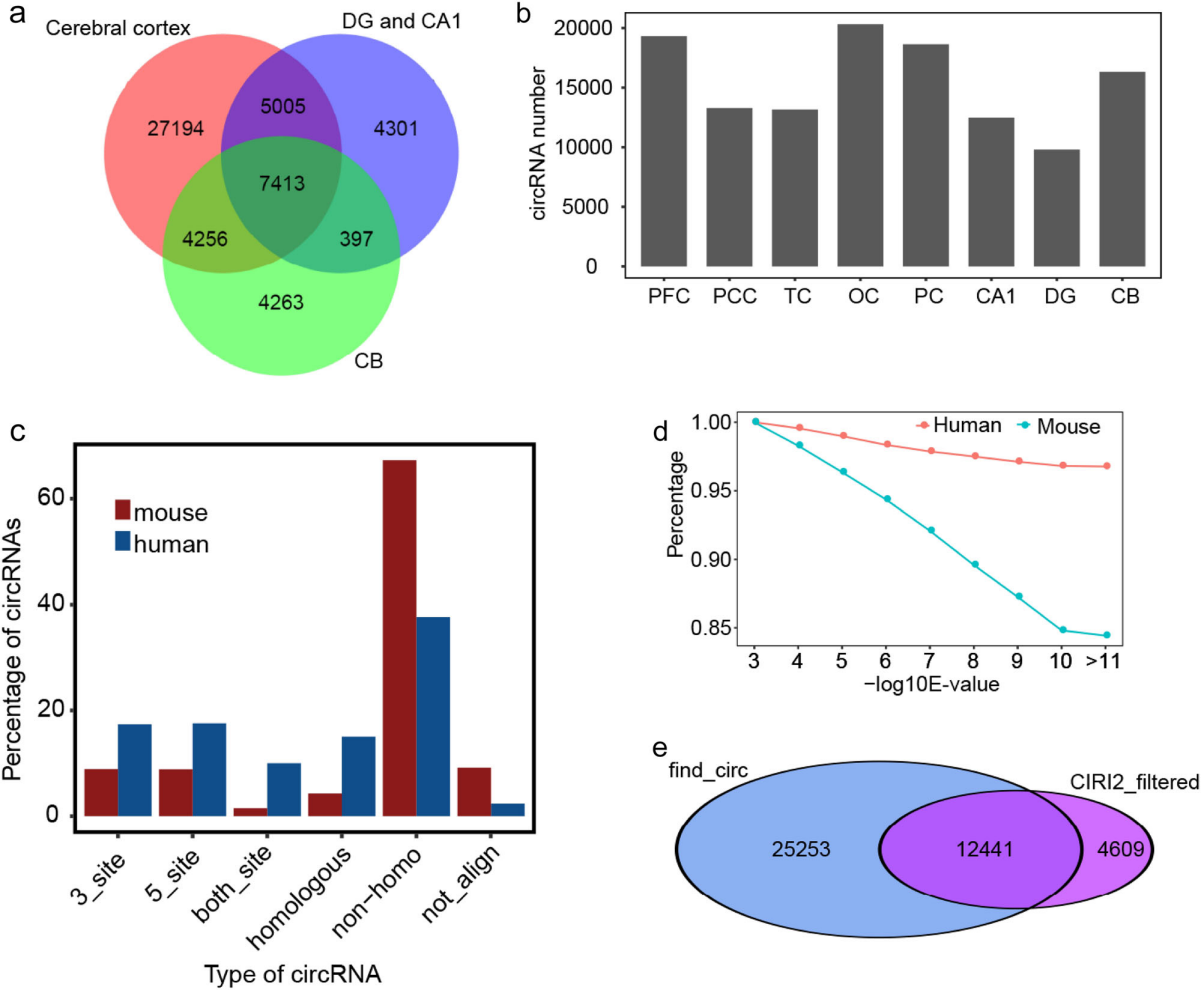
**The conservation of circRNAs in human and rhesus macaque**. **a** Venn diagram showed the overlapped result of circRNAs detected in three brain regions. **b** Bar plot of circRNA numbers detected in the eight brain areas. **c** Conservation analysis showed the higher conservation ratio between macaque and human compared with that of mouse, indicating the higher conservation between primates. **d** Linear plot of the decreased number of circRNAs by increasing the alignment threshold E-value by BLAST, which showed that circRNAs sequence character between human and macaque is more conserved than mouse. **e** Venn diagram showed the overlapped circRNAs between find_circ method and CIRI2 method after filtering

Next, we characterized the patterns of circRNA expression in the eight brain areas. Interestingly despite more specific circRNAs were detected in cerebral cortex compared with other regions (Fig. 2a), CA1 and DG showed less enriched for circRNA expression (Fig. 2b). To understand the features of circRNA expression in macaque brain, we plotted the abundance of circRNAs in each of totally sex- and age-matched 32-paired samples (Supplementary Fig. S2a). Indeed, the highly varying and dynamic expression pattern may be due to the low abundance of numerous circRNAs or the natural cell-type-specific feature of circRNAs^24^. To further explore the conservative property of circRNAs, we downloaded 140,790 and 16,444 circRNAs for human and mouse, respectively, from circBase^41^ and performed a comparative analysis. By utilizing the LiftOver software, we systematically mapped the human and mouse circRNAs to the macaque genome according to the genomic locus. With strict threshold, significant more macaque circRNAs find their homologies from human than mouse (Fig. 2c), indicating the better conservation of circRNAs between primates. Nevertheless, about 40% circRNAs could not be mapped to human circRNAs database, implying the inherent divergence feature of circRNAs. We also validated the homology analysis by blast method. Among the macaque circRNAs that have homology to human or mouse, 17,190 circRNAs (32.54%) were identified in all three species. The homologues between macaque brain circRNAs and the human circRNAs were much higher by increasing the blast stringency compared with the homologues between macaque and mouse (Fig. 2d).

To eliminate the influence of low expressed circRNAs, we filtered circRNAs expressed only in sporadic samples with low abundance (see methods for detail thresholds). Finally, we reserved a total of 17,050 unique circRNAs from the eight brain compartments, originated from 4902 host genes. 12241 circRNAs (73%) from these filtered circRNA sets were detected by find_circ (Fig. 2e). We then used the 17050 circRNA set to perform the following analysis, and searched the functions of these filtered circRNA host genes. Important neuronal pathways were enriched from the KEGG pathway (Supplementary Fig. S2b), such as Long-term potentiation, Glutamatergic synapse, Dopaminergic synapse, and Synaptic vesicle cycle. We also investigated these genes from the Gene Ontology (GO) database, and found that they also participated in the protein phosphorylation and catabolic process (Supplementary Fig. S2c). This analysis could provide a new perspective to investigate these circRNA-originating genes.

### Cerebellar circRNAs are distinct from that of other areas by spatial analysis

CircRNAs are enriched and differentially expressed in the mammalian brain^30,31^. To further define an overview of spatial patterns of circRNA expression in rhesus macaque brain, we explored circRNA expression modules by co-expression network analysis of WGCNA^42^. Cubic power was chosen as the soft threshold to calculate block wise modules (Supplementary Fig. S3a). This network identified 38 main circRNA expression modules (including module 0), each represented by a characteristic expression pattern (Supplementary Fig. S3b). CircRNAs number in each module ranged from 39 to 1652. Surprisingly, we found the correlation coefficient values were quite small as a whole from the dendrogram and correlation heatmap of the module eigengenes (Supplementary Fig. S3c). However, five modules (darkgrey, magenta, darkturquoise, lightcyan, and paleturquoise) were clustered together and showed high correlation between each other. Other modules were very distinct from each other, and the distance between the specific modules was very high (more than 0.8, Supplementary Fig. S3c).

**Fig. 3:**
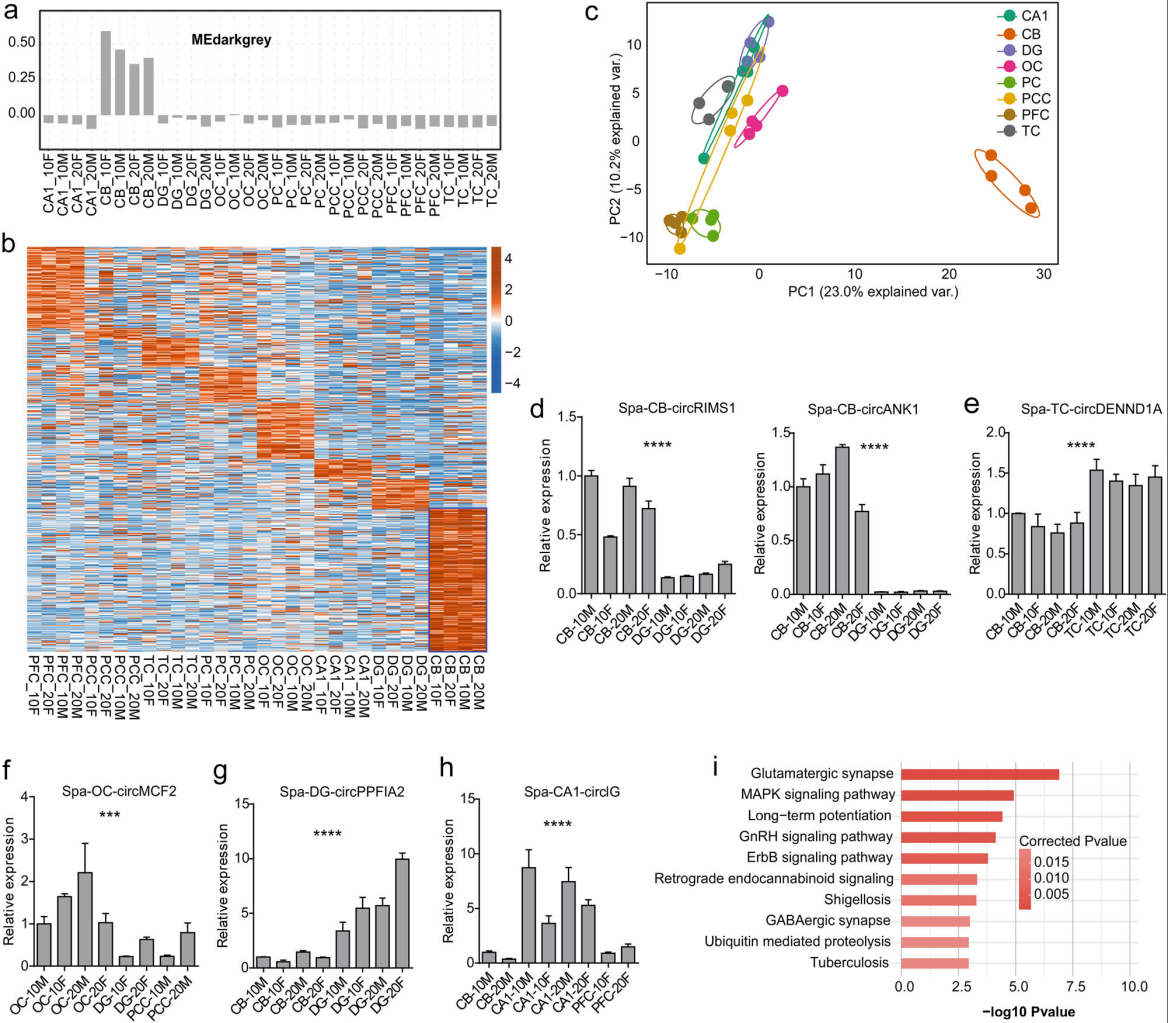
**Cerebellar circRNAs were enriched and distinct from that of other areas.** **a** Bar plot of the eigengene values for the darkgrey module, which exhibited CB enriched expression pattern compared with other samples. **b** Heatmap of spatial-specific circRNA expression level. CircRNAs were sorted by the spatial-specific order from PFC, PCC, TC, PC, OC, CA1, DG, and CB. CircRNAs from CB were prominent with blue frame. **c** PCA plot for the spatial-specific circRNA expression level. CB samples were distinct from other brain regional samples by PC1. **d**, **e** Bar plot presentation for the RT-qPCR results showed three selected circRNAs which were significantly enriched in CB and TC samples (Linear RNA of *GAPDH* was used as internal reference, and relative value of each sample was normalized by the first sample in each panel. *P-value < 0.05; **P-value < 0.01; ***P-value < 0.001; ****P-value < 0.0001, *t*-test. The star number represented the significant degree of expression between the two brain regions in each panel. The same as the following figures). **f**–**h** The same with (**d**) but for enriched circRNAs from OC (left), DG (middle), and CA1 (right). CA1 enriched circRNA came from intergenic region. (**i**) Bar plot of the top 10 enriched KEGG pathways of spatial-specific circRNA host genes

To further validate the above finding, we utilized other methods to explore spatial-regulated circRNAs in brain. By selecting circRNAs that have consistent higher expression level in one area than other areas using the *t*-test method (*p*-value < 0.01), we obtained 488 spatial-specific circRNAs for eight brain areas. Bar plot of eigengene values showed the special enrichment of CB region for the above five close modules (Fig. 3a, and Supplementary Fig. S3d). Expression pattern of other modules showed distinctly high level in one or two samples from different brain areas, which did not exhibit the regular expression pattern. Despite the fact that circRNA expression was more tissue- and cell-type specific than mRNA expression, our finding reflected that there existed circRNAs which possessed consistent and distinct expression patterns for the cerebellum region.

Among them, we found the CB region occupied the most quantitative circRNA numbers (177 enriched circRNAs, 36.27%), and their expression level was consistently high in the four-paired CB samples (blue frame in Fig. 3b). This phenomenon supports the above discovery that circRNAs in CB were abundant and distinct from other regions. Moreover, from another previous study on lncRNAs using the same biological samples, we also obtained the similar results that both lncRNAs and mRNAs from CB was distinct from other regions^40^. Spatial-specific circRNA groups of hippocampus regions (CA1 and DG, total 64, 13.11%) were the smallest circRNA sets compared with cerebellum and cerebral cortex (Fig. 3b). The spatial-specific circRNAs number from each neocortical region varied mildly from 13 to 98. PCA method was performed to assess the spatial expression patterns of these circRNAs. As shown in Fig. 3c, CB was also dramatically separated from all other regions, and other region samples exhibited similar pattern on the first principal component (PC1). To independently test these results, we selected tens of spatial-specific circRNA candidates with different expression levels. For five CB specific circRNAs, all of them were validated to be expressed higher in CB (Fig. 3d, and Supplementary Fig. S3e1-3). We also validated specific circRNAs from other brain areas. For example, circDENND1A (*p* = 0.05) for TC area (Fig. 3e); circMCF2 (*p* = 0.01), circCD99L2 (*p* = 0.04), circSMAD5 (*p* = 0.04), and circCAMK1 (*p* = 0.01) for OC area (Fig. 3f, and Supplementary Fig. Sf1-3); circMERTK (*p* = 0.05), and circGABRA4 (*p* = 0.03) for PC area (Supplementary Fig. S3g1-2); circPPFIA2 (*p* = 0.03) for DG area (Fig. 3g) and an intergenic circIG (*p* = 0.02) for CA1 area (Fig. 3h). This solid validation supports the conclusion that spatial-specific circRNA expression pattern exists in the macaque brain samples. We also analyzed the function of host genes for spatial-specific circRNAs. Enriched KEGG pathways for all spatial-related circRNA host genes were performed. Compared with the total circRNA host genes (Supplementary Fig. S2b), spatial-specific circRNAs yielded functional pathways more related to various signaling pathways related to brain development and function (Fig. 3i). Spatial classification of circRNAs revealed that plentiful circRNAs which were originated from the brain function-related host genes were distinctly expressed in the cerebral cortices.

### A sex-related circRNA expression found in rhesus macaque brain

**Fig. 4:**
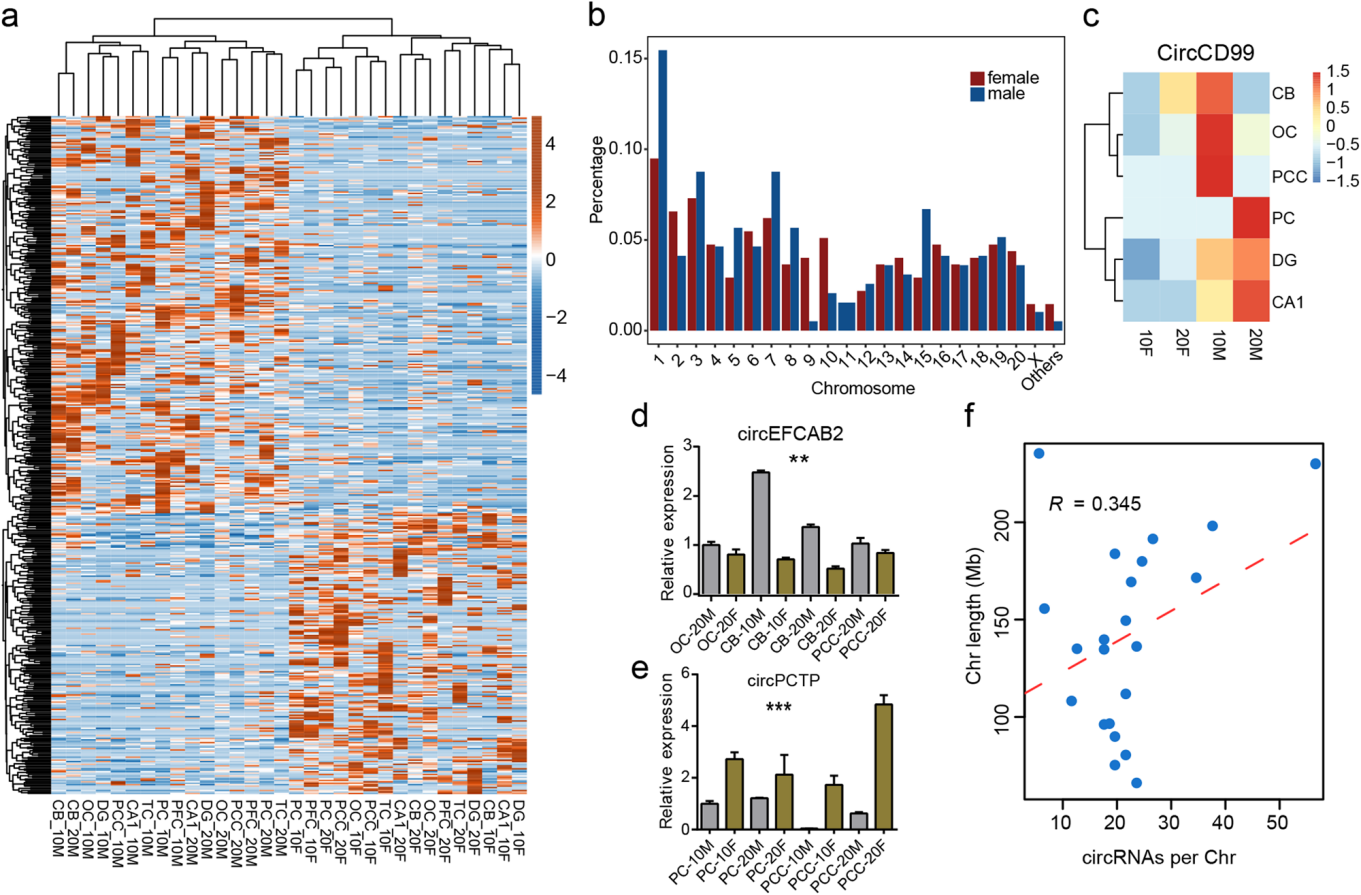
**Sex-biased circRNA analysis showed more male-biased circRNAs**. **a** Clustering heatmap of sex-biased circRNA expression levels. Top-left circRNAs were male-biased circRNAs, and bottom-right circRNAs were female-biased circRNAs. **b** Bar plot of the sex-biased circRNAs number from each chromosome. **c** Clustering heatmap of the circCD99 expression level, showing the male-biased pattern. **d**, **e** Bar plot presentation for the RT-qPCR results showed the male-biased circEFCAB2 (top panel, three brain areas) and female-biased circPCTP (bottom panel, two brain areas). The star number represents the significant degree of the expression between male (M) and female (F) samples, which were distinguished by different colors. The star numbers have the same meaning as shown in Fig. 3d, e. **f** Dot plot represents the positive relationship between sex-biased circRNA number and chromosome length (Mb). “*R*” was the Pearson correlation coefficient. Dashed red line was the regression line

From previous studies, sex-biased expression of protein coding genes has been reported in human brain^43,44^, but the sex-biased expression pattern for circRNA has not been reported. To further explore the impact of specificity of circRNA regulation, we screened sex-specific circRNAs from a different aspect regardless of the spatial and age regulation. By statistically identifying circRNAs differentially expressed between all male and female brain samples, a total of 468 sex-biased circRNAs containing 194 female-biased and 274 male-biased were obtained (Fig. 4a). A little more sex-biased circRNAs were detected by permutation test (Supplementary Fig. S4a). Highly overlapped circRNAs implied the consistent sex-biased circRNA results. Although no sex-biased circRNAs were detected from the Y chromosome, a larger fraction (58.54%) of male-biased circRNAs was detected, which was consistent with previous findings of mRNAs from human^43^. We observed more male-biased circRNAs (four circRNAs) were found from X chromosome than that from female (two circRNAs), but the total percentage of sex-biased circRNAs from X chromosome showed no enrichment (Fig. 4b). The circCD99, whose host gene CD99 was validated as male specific from Weickert et al.^44^, was also calculated as male specific from X chromosome (Fig. 4c). The similar male-biased expression patterns were found from circPREX1 and circTSPAN15 (Supplementary Fig. S4b-c), whose host genes were treated as male specific by Kang et al.^43^. We further validated several circRNAs showing sex-biased expression pattern. For example, circEFCAB2 was for male-biased expression pattern, circPCTP and circZNF484 were for female-biased pattern (Fig. 4d, e, and Supplementary Fig. S4d). Sex-biased circRNA number in each chromosome exhibited moderately positive correlation with the chromosome length (*R* = 0.345, Fig. 4f). The positive correlation and the consistent bias with host mRNAs of sex-specific feature indicated that sex-specific circRNAs showed no allosome bias and may be influenced by their host linear RNAs. We then analyzed the expression pattern of host genes of these sex-specific circRNAs. However, spatial factor was more pronounced than the sex factor for these genes (Supplementary Fig. S4e), implying the distinct expression patterns of circRNAs and their host genes. To investigate the functional identity of circRNAs in their sex-biased features, functional enrichment analysis of host mRNA genes with each of the sex-related circRNA was performed to allow us to further annotate these circRNAs. From the biological process (BP) term of the GO database, several terms related to the signal transduction, protein modification and transport were significant enriched compared with the background, relating to various physiological and pathological states of primate brain in a sex-biased manner (Supplementary Fig. S4f). Cellular component (CC) terms from GO database revealed that host genes of these circRNAs were mainly distributed in cytoplasm, nucleoplasm microtubule, synapse, postsynaptic membrane, and density (Supplementary Fig. S4g). KEGG pathways were also obtained for all host genes of sex-biased circRNAs, which yielded distinct functional pathways including RNA degradation, Ubiquitin mediated proteolysis, Bacterial invasion of epithelial cells, Fc gamma R-mediated phagocytosis, related to various physiological and pathological states of primate brain in a sex-biased manner (Supplementary Fig. S4h).

### Age-related accumulation of circRNAs in the eaged rhesus monkey brain

**Fig. 5:**
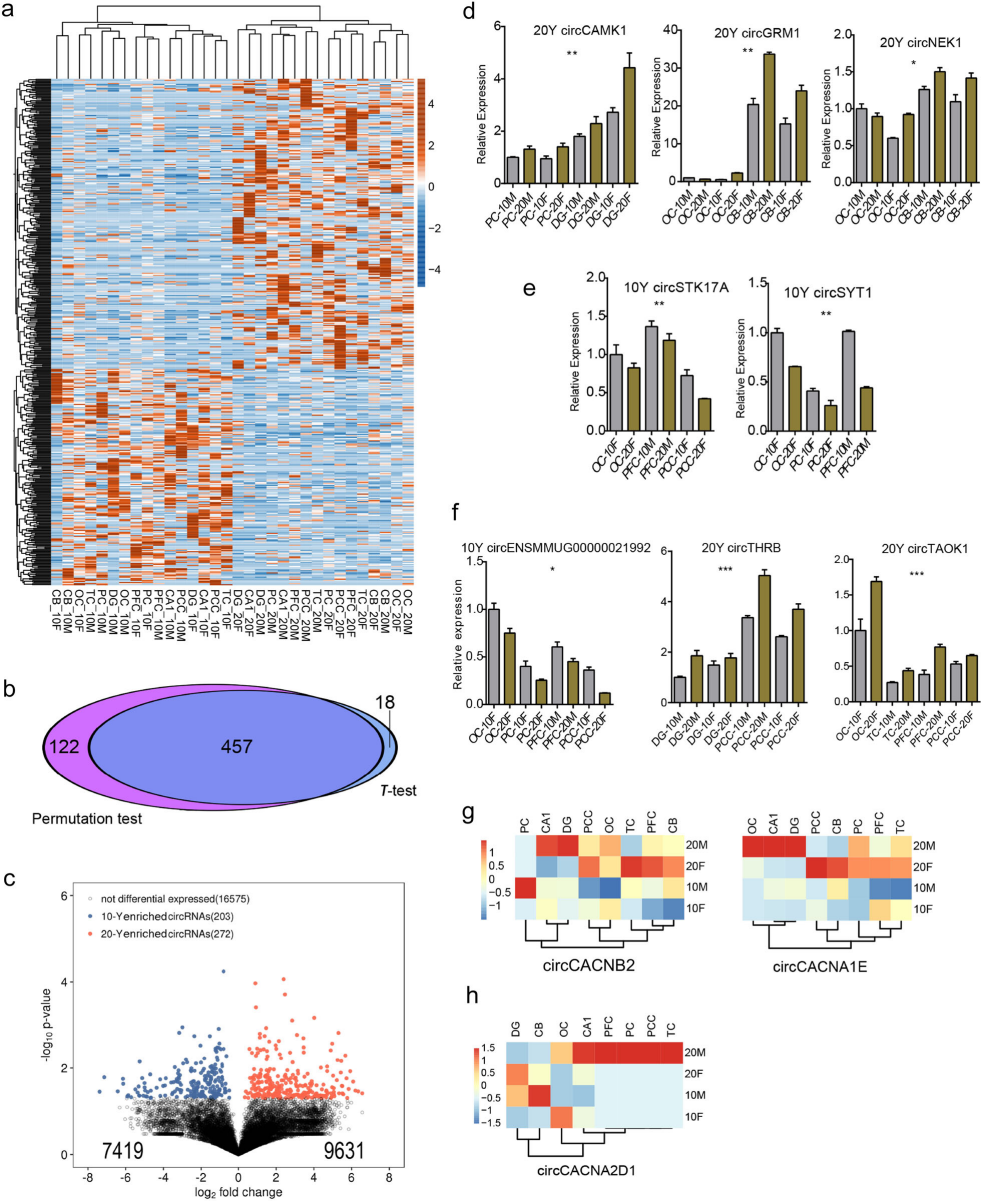
**Characteristics of age-related circRNAs was identified in macaque brain.** **a** Clustering heatmap of aging-related circRNA expression levels. Top-left represents highly expressed circRNAs at 10-year-old, and bottom-right represents highly expressed circRNAs at 20-year-old. **b** Venn diagram showing the overlapping result of aging-biased circRNAs detected by permutation test and *t*-test. **c** Volcano plot representing the fold changes and significant *p*-values between 10 y and 20 y samples. Blue and Red dots represent circRNAs significantly more expressed in the 10 y and 20 y samples (*p*-value < 0.05), respectively. The numbers in the bottom indicate all circRNAs more expressed in 10 y (7419) and 20 y (9631) samples, (|log2 Fold Change| > 0). **d** Bar plot presentation for the RT-qPCR results showed three age-biased (20 y biased) circRNAs. Four brain regions were selected to perform the experiment. The star number represents the significant degree of the expression difference between 10 y and 20 y samples distinguished by different colors. It is the same as in (**d**) and (**e**). The star numbers have the same meaning as shown in Fig. 3d, e. **e** Bar plot presentation for the RT-qPCR results showed two age-biased (10 y enriched) circRNAs. Four brain regions were selected to perform the experiment. **f** Bar plot presentation for the RT-qPCR results showed three age-biased circRNAs closing to the threshold. Six brain regions were selected to perform the experiment. **g**, **h** Clustering heatmaps of circCACNB2, circCACNA1E, and circCACNA2D1 expression level variations were determined during aging, which showed increased expression in 20 y

Given that the high abundance of circRNAs in the mammalian brain^30^, the increasing expression level during aging in fruit fly central nerve system^21^ and photoreceptors^45^, and the global age-accumulation in *C. elegans*^46^ and mouse brain^21,32^. However, decreasing expression pattern of circRNAs during aging was also validated in monkey skeletal muscle^33^, which has similar aging points with our study. Therefore, we asked whether the dynamics expression pattern of circRNAs in primate is associated with aging-related changes in brain function and structures. We screened aging-related circRNAs from the aspect regardless of the sex and spatial regulation. We also used *t*-test method to identify circRNAs differentially expressed between 10 years and 20 years brain samples. A total of 475 age-biased circRNAs, containing 272 20-year specific and 203 10-year specific, were found during brain aging (Fig. 5a). 579 age-biased circRNAs were detected by permutation test (*p*-value < 0.05), showing a highly overlapping ratio (79%, 457/579) with *t*-test result (Fig. 5b). Volcano plot of all the circRNAs also showed a globally biased expression toward the 20 y macaque brains, showing that 9631 (56.49%) were higher expressed (|log_2_ Fold Change| > 0) in the 20 y while 7419 (43.51%) in the 10 y samples (Fig. 5c), although most of them did not reached a significance. From the above result, more aging specific circRNAs were observed in the 20 years samples compared with the 10 years sample, illustrating that circRNA expression level increased during aging. Meanwhile, only 73 age-related circRNAs were found by strict criteria (*p*-value < 0.01). This phenomenon indicated that a few circRNAs exhibited consistent age-related characters across all brain regions. This finding was consistent with previous finding from fruit fly^21^ and mouse^32^ brain regions, but inconsistent with that from monkey muscle tissue^33^, implying the distinctly age-related functions of circRNAs in brain regions.

We then selected several circRNAs to validate the age-related characters by performing RT-qPCR experiment. CircCAMK1, circGRM1, and circNEK1 showed 20-year-specific pattern by RT-qPCR (Fig. 5d). CircSTK17A and circSYT1 showed 10-year-specific patterns (Fig. 5e). We also selected some circRNAs that did not meet the threshold (*p*-value < 0.05) but were close to the threshold for RT-qPCR validation. One and six candidate circRNAs were further validated for 10- and 20-year-old specific circRNAs, respectively. Figure 5f exhibited three circRNAs of them for presentation. We then investigated expression level of circRNAs whose host genes were voltage-gated calcium channel superfamily that are involved a variety of calcium-dependent signaling, including synaptic plasticity, muscle contraction, hormone or neurotransmitter release, and gene expression. CACNB2, CACNA1E, and CACNA2D1 were members of the superfamily. Surprisingly, all CACNB2-, CACNA1E-, and CACNA2D1*-*derived circCACNB2, circCACNA1E, and circCACNA2D1 showed higher expression level in the 20 y than that of 10 y (Fig. 5g). This overall changes in circRNA expression during aging indicates that circularization is not only accompanying anatomical structure commitment, but is also likely important for deregulated brain function during aging, because many circRNAs are down- or upregulated at the time points of brain aging

### Differential correlations between age-related circRNA and host mRNA expression

**Fig. 6:**
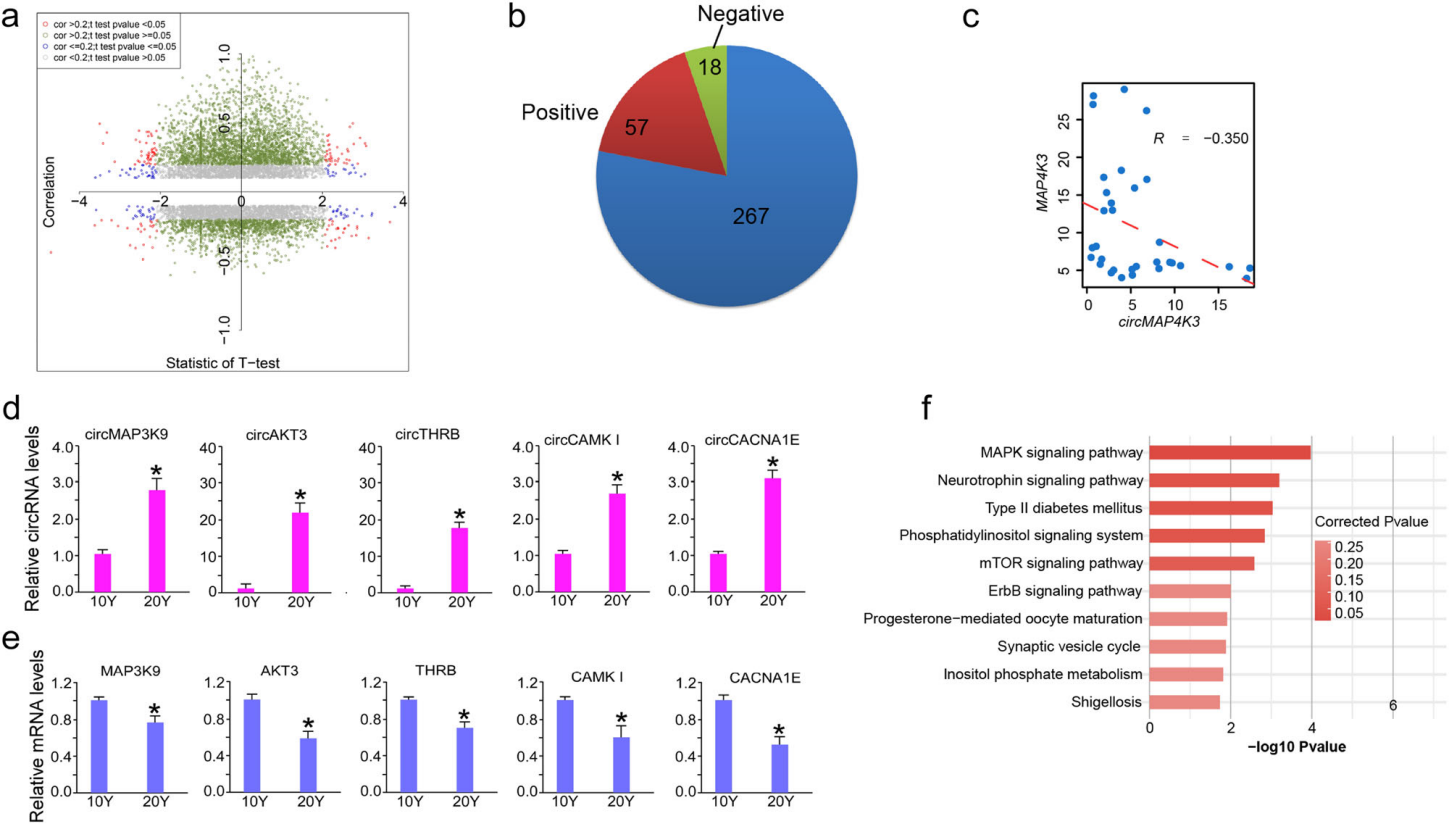
**Age-related circRNAs showed correlation with host mRNA production**. **a** Dot plot representation of the relationship between age-related circRNA expression pattern and correlation with host mRNAs for all circRNAs in PCC. X axis represents the *t*-statistics value between 10 y and 20 y samples; Y axis represents the correlation coefficients between circRNAs and host genes. **b** Pie chart showing the percentage of circRNAs that were correlated with host mRNAs among the aging-related circRNAs. CircRNAs correlated with host mRNAs were separated as positive and negative correlation parts. **c** Dot plot represents the negative relationship between circRNA and host mRNA expression. CircMAP4K3 were chosen as examples. “*R*” was the Pearson correlation coefficient. Dashed red line was the regression line. **d**, **e** Total RNAs were extracted from frozen 10 y and 20 y macaque hippocampal tissues, and performed quantitative RT-qPCR for validation age-related circRNAs, and their host mRNA expression. Data are presented as mean ± SD, *n* = 3 technical replicates. *p < 0.05, unpaired *t* test. *GAPDH* was used as the internal reference. **f** Bar plot of top 10 KEGG pathways for the circRNA host genes which were correlated with circRNAs

While previous studies suggest that relative levels of circRNAs and linear transcripts from the same gene can differ between cell types as well as neuronal differentiation due to their differential degradation and/or production^24,30^. To assess this phenomenon in primate brain during aging, we calculated the Pearson correlation coefficients (PCCs) between circRNAs and their host mRNA expression. The linear mRNA transcriptional data were extracted from ours another study^40^ using poly(A)-enriched RNA-seq method with the same biological samples (see methods). After filtering, we observed that 961 circRNAs-mRNA pairs (19.6%) were correlated at a threshold of absolute 0.3 PCC (Fig. 6a, green and red dots), including 797 positive and 164 negative correlated pairs. From the total pairs between circRNAs and host genes in Fig. 6a, positive correlation pairs were dominant. Consistent with the previous studies, our data revealed that overall, most circRNAs (~80%) expression have no significant correlation coefficient with their host mRNAs. However, 20% of the positive and negative correlation between circRNAs and their host transcripts indicates the prevalence of the cooperative and competition expression relationship between macaque brain circRNAs and host mRNAs.

We then investigated the relationship between the aging-related circRNAs and their host mRNA. Out of the 342 aging-related host genes, 75 was also found in the correlated pairs (*p*-value = 0.034, hypergeometric test, Fig. 6b). Noticeably, despite 57 out of the 75 showing e positive correlation, we also found 18 negative correlated pairs. This result is correlated with the total phenomenon that circRNAs were more probably positively correlated with host transcripts. Negative correlation pairs could be regard as the competition relationship between circRNAs and host genes. We showed that circMAP4K3 and its host mRNA pairs were the competition relationship as examples (Fig. 6c). We further validated age-related circRNAs, cicrMAP3K9, cicrAKT3, cicrTHRB, cicrCAMK1, cicrCACNA1E, and their host mRNA expression in rhesus macaque hippocampal tissues of 10 y and 20 y, showing that changes in circRNA expression inferred from sequencing data overall correlate with qPCR measurement (Fig. 6d, e). To identify function of circRNAs showing correlated expression with host mRNA genes, we analyzed enriched GO terms and KEGG pathways obtained from all mRNA genes correlated with circRNAs. Several clusters yielded distinct functional pathways related to various physiological and pathological states including MAPK signaling pathway, neurotrophin signaling pathway, Type II diabetes mellitus, Phosphatidylinositol signaling system, mTOR signaling pathway, ErbB signaling pathway, Synaptic vesicle cycle (Fig. 6f).

### Upregulation of calcium channel gene-derived circRNAs found in the aged brain

Brain aging was attributed to many dysfunction of brain including loss of calcium homeostasis and synaptic plasticity^4,47,48^. To get a view of whether age-related circRNAs are devoted in some way to contribute to the neurobiology of aging, we selected 41 circRNAs containing 7 calcium signaling, 16 receptors, 18 protein kinase host genes (Supplementary Fig. S5a). Then we screened these circRNAs from aged-related circRNAs identified from the above part (*t*-test, *p*-value < 0.05). Strikingly, 30 of these circRNAs (73.17%, *p*-value = 2.14e−38, hypergeometric test) were found from the age-related circRNAs (*p*-value < 0.05) and all of them showed age-biased expression pattern. We then analyzed the correlation between these age-related circRNAs and their host mRNA expression using our previous RNA-seq data (Supplementary Fig. S5b)^40^. Eleven circRNAs (circCACNA1E, circMAP2K5, circERBB4, circLATS1, circGABBR2, circBMPR2, circGRIA1, circCACNA2D1, circMASTL, circMAPK1, and circCACNB2) were overlapped, including 9 negative correlated and 2 positive correlated.

**Fig. 7:**
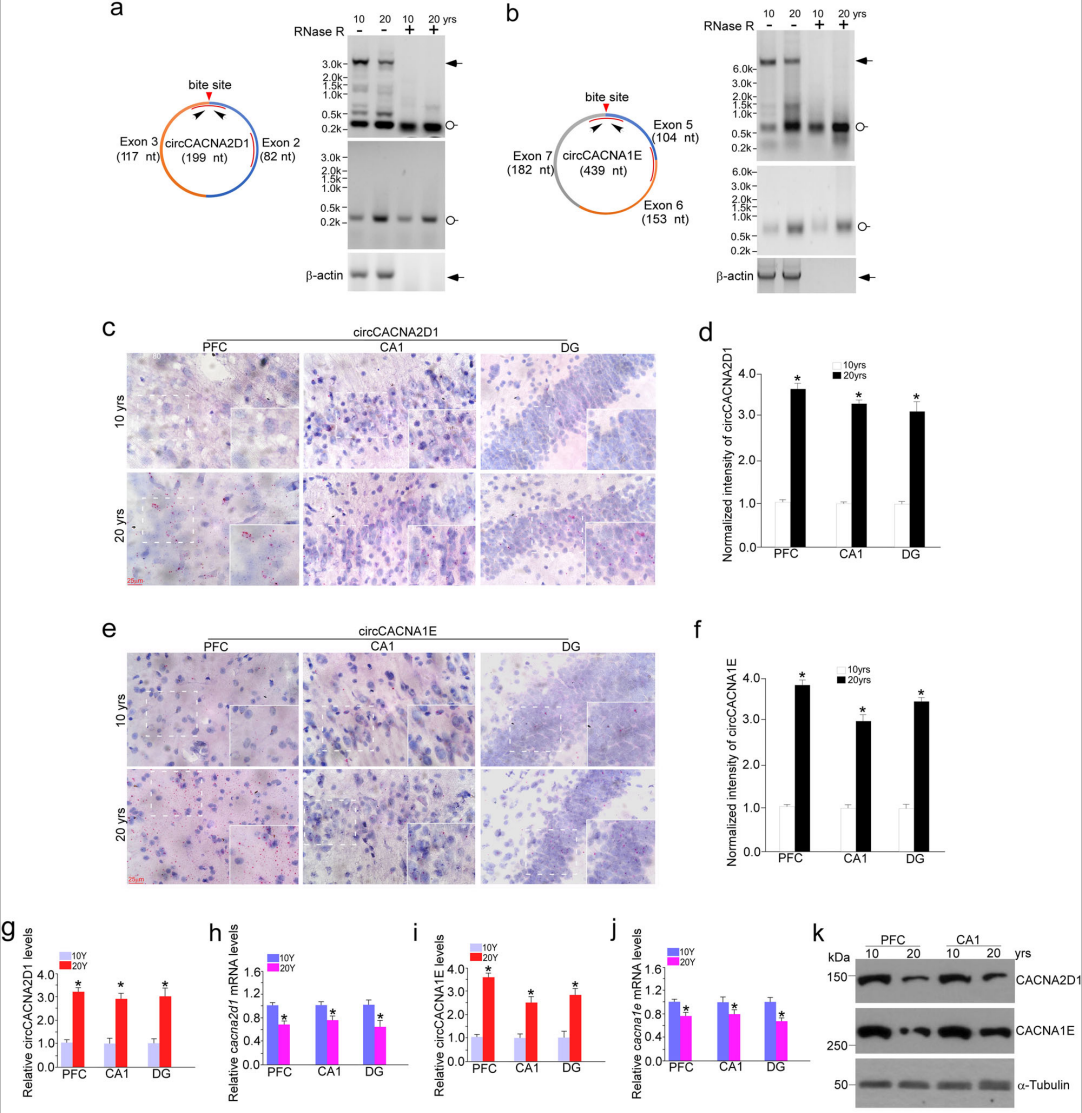
**CircCACNA2D1 and circCACNA1E expression negatively correlated with host mRNA production over the aging states.** **a**, **b** Representative northern blots of *cacna2d1* and *cacna1e* linear and circular transcripts. Total RNAs were extracted from 10 y and 20 y macaque frozen CA1 tissues, and digested with or without RNase R. Northern blots were performed on the basis of hybridization with the specific Digoxin-labeled junction site-targeting (lower panel) or single exon-targeting (upper panel) circRNA probes showing that increased levels of circular transcript expression were age-related. K denotes 1000 nt. The blots are representative of replicates of three independent experiments. β-actin mRNA was the negative control. **c**, **e** PFC and hippocampal ten-micron cryostat sections of 10 y and 20 y sex-matched macaques were performed BASEScope with specific 1zz junction site-targeting probes against circCACNA2D1 and circCACNA1E, respectively (red dots with nuclei counterstaining by hematoxylin). Scale bar, 25 μm. White squares represent high magnificent images. The images are representative of replicates of three independent experiments. **d**, **f** Relative intensities of subcellular localization BASEScope signals of circRNAs illustrated in (**c**, **e**) were quantified by use of image J software. Data are present as mean ± s.e.m. (*n* = 60–81 cells per group). *p < 0.05, unpaired *t* test. **g–j** Total RNAs were extracted from 10 y and 20 y frozen postmortem macaque brains at indicated regions, and performed qPCR for validation circCACNA2D1 and circCACNA1E, and their host mRNA expression. Data are presented as mean ± SD, *n* = 3 technical replicates. *p < 0.05, unpaired *t* test. **k** Protein extracts from 10 y and 20 y rhesus macaque frozen PFC and hippocampal CA1 tissues were immunoblotted with CACNA2D1 and CACNA1E antibodies. α-Tubulin was loading control. The blots represent one of three independent experiments

Brain aging leads to a decrease in several voltage operated calcium channel (VOC) mRNA expression^49,50^. Due to the important function of voltage-gated calcium channels in the maintenance of calcium homeostasis and signal transmission of neuron cells^51^, we decided to examine whether increased expression levels of two age-related calcium channel gene-derived circCACNA2D1 and circCACNA1E were associated with the decreases of their host calcium channel (VOC) mRNA expression over the aging states. First, using northern blot on the basis of hybridization with specific circRNA junction site-targeting probes we examined two highly abundant circRNAs in the brain, circCACNA2D1 (L-type Ca^2+^ channel gene) and circCACNA1E (R-type Ca^2+^ channel gene) expression in macaque hippocampal CA1. Strikingly, both of them showed significant increases in the 20 y samples (Fig. 7a, b). Next, using BASEScope (circRNA-specific in situ hybridization) with circRNA junction site-targeting probes we further verified the increased expression levels of circCACNA2D1 and circCACNA1E in macaque brains of 20 y compared to that of 10 y (Fig. 7c–g, and Supplementary Fig. S6). We further examined circCACNA2D1, circCACNA1E and their host mRNA expression in the frozen PFC, CA1 and DG tissues from 10 y and 20 y rhesus macaque postmortem brains. Indeed, in consistent with BASEScope results, the data of qPCR revealed a negative correlation between the two circRNAs and their host mRNA expression over aging states (Fig. 7g, i). Indeed, IHC data indicated that the host mRNA expression showed a decrease in the aged macaque brains (Supplementary Fig. S7a-d). We further assayed the protein levels of CACNA2D1 and CACNA1E in macaque brain PFC and CA1, and found them to be dramatically decreased in 20-year old macaque (Fig. 7k).

To further explore whether circCACNA2D1 and circCACNA1E negatively regulate host mRNA expression, first, we examined and validated the levels of circCACNA2D1 and circCACNA1E expression in cultured fetal macaque hippocampal neurons using northern blot on the basis of hybridization with circRNA exon probes (Supplementary Fig. S8a-b). As expected, a significant increase in circCACNA2D1 and circCACNA1E expression was found in P28 cultures in contrast to P14 cultures. Nonetheless, the cultures with siRNAs infection at P14 showed a dramatic decreased expression in P28. Next, we examined levels of *cacna2d1* and *cacna1e* gene expression using RT-qPCR, immunofluorescence and western blot (Supplementary Fig. S8c-h). Empirically, the similar phenomena revealed that knockdown of circCACNA2D1 and circCACNA1E could reverse the decreased levels of calcium channel gene expression.

## Discussion

In this study, using RNAs with depletion of linear RNAs by RNase R from rhesus macaque multiple brain compartments we sequenced and analyzed circRNAs in a quantitative manner. While circRNA identification mostly relied on genome annotation^23,30,52,53^, we used the updated circRNA identifier software CIRI2 and find_circ^23,36^ to identify circRNAs with remarkably balanced sensitivity, reliability, duration, and RAM usage. CIRI2 also does not rely on exon annotations or assumption of canonical splice sites, and can thus identify circRNAs derived from previously unannotated exons and transcripts, as well as the intergenic regions. This allowed us to identify circRNAs in rhesus macaque brain, which, up-to-date, has relatively incomplete annotations of transcriptome and functions, particularly in the aged brain. Using this approach, 17,050 well expressed circRNAs were identified in rhesus monkey brain. Functional analyses and exploration of two specific calcium channel gene-derived circRNAs, suggesting that age-related circRNA biogenesis regulates its host mRNA production, and is potentially involved in the biological process of brain aging.

In this study, we sacrificed eight macaques in total, two females and two males at 10-year and 20-year ages. After extracting total RNAs, the two biological replicates were mixed together to prepare libraries and circRNA-seq, which should effectively eliminate the problem of individual biases. Furthermore, we have obtained circRNA-seq data from eight different regions of each mixed pair of animals. Therefore, a total of 32 circRNA-seq data were generated to analyze the spatial-, sex- and aging-regulated circRNAs. For each set of statistical analysis, there were either 4 or 16 circRNA-seq data in each compared group, which should be enough to avoid the interference of experimental variations and to yield confident results. We analyzed sex-regulated and aging-regulated circRNAs by both permutation and *t*-test, which yielded highly similar results. Taken together, the experimental approaches applied should be adequate to support the related conclusions. Meanwhile, we are aware of the limitations of the conclusions drawn from an aging trend with only 2 time points. Future studies are needed to include more time points during macaque aging.

We observed that circRNAs are more conserved between human and macaque than that between mouse and macaque (Fig. 2c). This led us the hypothesis that human specific circRNAs may play more important roles in perception and learning. Moreover, we analyzed the common of circRNAs detected in macaque and human, 23,921 (45.28 %) of the circRNAs identified in macaque PFC and cerebellar cortex were also expressed in human PFC and cerebellar cortex. This further suggests that circRNAs are well conserved, and our observation that 13.67% overlap between macaque and human circRNAs may prove to be an underestimate. Notably, the exon sequences around head-to-tail junctions detected in both macaque and human are extremely conserved, strongly suggesting their potential functional relevance.

While circRNAs are ever thought as side products of pre-mRNA splicing^54^, recent studies have suggested that circRNAs have biological functions based on their strong tissue- and cell-type-specific expression^31^. For instance, brain-specific circRNAs are expressed from gene loci that also code for proteins take part in synapse-related functions^30^. In the present study, we have extracted circRNAs showing spatial-specific, sex-biased, aging-biased, and relationship with host mRNA characters. In line with former studies, we found most of the circRNAs (~90%) showed none of this characteristic and their expression pattern seemed to be random. Nonetheless, there is also over one thousand circRNAs with these features. Among which about 3% circRNAs (475 out of 17,050) participates in the biology of brain aging. To investigate if changes in circCACNA2D1 and circCACNA1E expression are associated with their host calcium channel gene mRNA expression, we detected and revealed a strong correlation between *c*ircCACNA2D1, circCACNA1E, and their host mRNA expression in rhesus macaque brain over the aging states. Further investigation may help to elucidate the role of increased circCACNA2D1 and circCACNA1E expression in the neurobiology of aging.

Overall, this is a first systematic analysis of circRNA profiles in nonhuman primate brain, and is also a first clustering of changes in circRNA expression during brain aging. We generated a highly qualitative novel resource for the neuroscience community. Variance decomposition analysis was used to obtain a quantitative assessment of the involvement of circRNAs in the biology of brain aging, independent of genetic influences. This deep characterization of molecular events is expected to substantially boost focused functional explorations of circRNA-mediated regulatory system, and an important step toward further elucidation of circRNA function underlining the biology of brain aging.

## Materials and methods

### Animals and samples collection

Frozen postmortem brain tissue samples from rhesus macaque were provided by Kunming Primate Research Center of the Chinese Academy of Sciences (KPRC). Brain regions were systematically collected from well-characterized rhesus monkeys born and raised at the KPRC in outdoor, 6-acre enclosures that provide a naturalistic setting and normal social environment. For circRNA-seq analysis, total RNAs from postmortem rhesus macaque brain specimens of two males and two females at each of stages representing adult (10-year-old) and old (20-year-old) were extracted. Extensive health, family lineage, and dominance information were maintained on all animals.

According to a widely used macaque brain atlas and brainmaps (http://www.Brainmaps.org), tissues spanning eight anatomically distinct regions were selected and collected from each specimen. The detailed information was described as below: the PFC was sampled at the main sulci, the PCC was sampled at the Brodmann’s area 23, the TC at the superior temporal gyrus, the PC at the middle sylvian fissure, the OC at the V1, and the cerebellar cortex was sampled at the cauda cerebellum. The hippocampus (including CA1 and DG) was also sampled. All the collected samples were washed with RNA later solution (AM7021, Ambion, USA) and put in freezing tubes to store at liquid nitrogen temperature.

All animal procedures were in strict accordance with the guidelines for the National Care and Use of Animals approved by the National Animal Research Authority (P.R. China) and the Institutional Animal Care and Use Committee (IACUC) of the Kunming Institute of Zoology of Chinese Academy of Sciences. The nonhuman primate cares and experimental protocols were approved by the Ethics Committee of Kunming Institute of Zoology and the Kunming Primate Research Center, Chinese Academy of Sciences (AAALAC accredited), and the methods were carried out in accordance with the approved guidelines.

### circRNA library preparation

Before the preparation of the circRNA-seq libraries, the total RNA samples for two males were mixed and those for females were mixed as well to eliminate the individual biases. Samples were collected from eight different brain regions, and consequently a total of 32 mixed samples from 10-year male and female, and from 20-year male and female were subjected to library preparation. For each sample, 10 μg of total RNA was used for cirRNA-seq library preparation. Total RNA was treated with RQ1 DNase (Promega) to remove genomic DNA. A large proportion of mRNA was captured by oligo dT, and remaining RNA was purified with Ampure XP Beads. Ribosomal RNA was depleted away by Ribo-Zero Magnetic Gold Kit (Human/Mouse/Rat, MRZG12324, Epicentre). Linear RNA (including residual rRNA, residual mRNA, and so on) was digested by RNase R. CircRNA was iron fragmented at 95℃ followed by end repair and 5′ adaptor ligation. The reverse transcription was performed with RT primer harboring 3′ adaptor sequence and randomized hexamer. The cDNAs were purified and amplified, then PCR products corresponding to 300–500 bp were selectively captured and quantified. Libraries were stored at −70 °C until used for sequencing. For high-throughput sequencing, the libraries were prepared following the manufacturer’s instructions and applied to Illumina NextSeq500 system with 150 × 2 paired-end type by ABlife Inc. (Wuhan, China).

### circRNA Prediction and filtering

After reads were obtained from sequencing, Cutadapt^55^ was used to trim adaptors (with 10% error rate) and low quality bases (less than 16). Reads that were less than 16nt in both ends were discarded. Before prediction, we used bwa^56^ method to align reads to the *Rhesus Macaque*^57^ reference genome in local mode (with parameters: -k 19-T 19). After alignment, CIRI2 software^36,58^ was used to predict circRNAs in macaque brain samples. Candidate circRNAs with no less than two head-to-tail junction reads were preserved. To test the precision and sensitivity of the CIRI2 software, we downloaded the raw data of the SH-SY5Y neuronal cells^30^. We aligned the reads to the human genome (GRCH38 version) and predicted the circRNAs with our prediction pipeline with the same parameters. Then we converted the published circRNAs locus from GRCH37 to GRCH38 by liftOver^59^. circRNAs which splice sites were detected in ±5 nucleotide interval around the predicted circRNAs start or end were treated as overlap circRNAs. At the same time, we re-predicted circRNAs with our data by utilizing the find_circ (version 1.2) software with default parameters^23^. Overlapping analysis was performed for the prediction results between CIRI2 and find_circ. CircRNAs are constituted by two parts by CIRI2: back splicing junction part and non-junction part. After obtaining the circRNAs, reads number across the back-splicing junctions was treated as the raw expression level of each circRNAs. After that, we filtered the circRNAs expressed only in sporadic samples with low abundance. Based on the back-splicing reads number of each circRNA, we set the following requirements as the filtering threshold:

1. No less than 3 back-splicing reads was required to define an expressed circRNA.
2. One brain area is defined as expressed only if no less than 2 samples satisfied the 1st requirement.
3. One circRNA is expressed only if no less than 2 areas were expressed.
4. If circRNAs cannot meet the 3rd requirement, the circRNAs must be expressed in only one brain area and the total expressed samples must be on less than 6 (20% of total samples).
5. If circRNAs cannot meet the 3rd and 4th requirements, the total back-splice reads number of this circRNA must be no less than 30.

To obtain a normalized expression between different samples, we used the DESeq normalization method^37^ to obtain the normalized expression level for circRNAs by dividing a size factor.

### Conservation analysis for macaque circRNAs

To analyze the homologous feature between macaque circRNAs and other mammalian species, we downloaded the human and mouse circRNAs sequence from the circBase^41^. Then we aligned the circRNA sequence to the human and mouse circRNAs by blastn with E-value 1e−3. Meanwhile, we also adopted the conservation calculation method in Rybak–Wolf et al.^30^, and performed this analysis among three species: macaque, mouse, and human.

### Characteristic analysis of circRNA expression

To find the characteristic of circRNA expression in macaque brain, we compared the circRNA expression pattern from multiple dimensions. First, we used the WGCNA method^42^ to classify circRNAs. Cubic power was chosen as the soft threshold to calculate block wise modules. The output is the circRNA modules according to their expression pattern. For each circRNA module, eigengenes was chosen to represent the expression pattern. Due to the low and dynamic characteristic of circRNAs compared with host mRNAs, we used unpaired *t*-test model to filtrate the spatial, aging and sexual-related circRNAs as described previously^43^. For spatial-regulaed circRNAs filtration, we compared one brain area with other areas by *t*-test and repeated this according the eight brain areas. For aging-regulated circRNAs filtration, we compared 10-year-old samples with 20-year-old samples. For sexual-regulated circRNAs filtration, we compared female samples with male samples. After comparison, we set *p*-value 0.05 as the threshold for filtration.

### Analysis of the relationships between circRNAs and host mRNAs

To explore the expression relationship of circRNAs and their host mRNAs, we used the poly(A) selected RNA-seq data from our another study^40^. The biological samples of the two studies were the same and total RNAs were extracted from the brain tissue parallelly. So, we could compare the expression level between circRNAs and host mature mRNAs. Based on the expression of each circRNA and host mRNA, PCC and *p*-value were obtained for each mRNA-circRNA pair. Then we filter the result by a given threshold, absolute PCC no less than 0.3 and *p*-value no more than 0.1. Besides the positive correlation pairs, negative pairs with correlation coefficient less than 0 were also included.

### RNA extraction and reverse transcription PCR

RNA was using PureLink micro-to-midi total RNA purification system (Invitrogen). RNA quantity and quality were evaluated by Nano Drop ND-1000 spectrophotometer (Nano Drop Thermo, Wilmington, DE) and RNA integrity was assessed by agarose gel electrophoresis. Specific divergent primers were designed to amplify the circular and linear Rhesus macaque transcripts. Semi-quantitative RT-PCR was performed with Superscript III one-step RT-PCR system with platinum Taq High Fidelity (Invitrogen). For quantitative real-time PCR, cDNAs were prepared by using oligonucleotide (dT), random primers, and a Thermo Reverse Transcription kit (Signal way Biotechnology). qPCR was performed with SYBR Green I Master (Roche, 04707516001) on Light Cycler 480 II. qPCR was performed in 10 μl reaction volume, including 2 μl of cDNA, 5 μl 2 × Master Mix, 0.5 μl of Forward Primer (10 μM), 0.5 μl of Reverse Primer (10 μM) and 2 μl of double distilled water. The reaction was set at 95 °C for 10 min for pre-denaturation, then at 95 °C for 10 s and at 60 °C for 60 s repeating 40 cycles. *GAPDH* was used as a reference. Both target and reference were amplified in triplicate wells. And the relative level of each circular and linear transcript was calculated using 2^−△△Ct^ method. All PCR primer sequences can be found in Supplementary Tables S3 and S4.

### Immunohistochemistry

For DAB/bright field staining, ten micron cryostat sections of macaque brain were pretreated in 0.3% hydrogen peroxide in methanol for 30 min to remove endogenous peroxidase activity, rinsed in tris-buffered saline (TBS), and then treated with 0.1 M citrate buffer in a microwave at sufficient power to keep the solution at 100 °C for 20 min. Sections were cooled in the same buffer at room temperature (RT) for 30 min and rinsed in TBS. Slides were incubated in 10% goat serum in PBS blocking solution for1 h at RT, after which primary antibodies CACNA2D1 (ab2864) and CACNA1E (ab63705) were applied to the sections that were then incubated at 4 °C overnight. The sections were washed three times in TBS before applying the secondary antibody (Vector Laboratories). Secondary antibody was applied for 1 h at RT. Afterwards, sections were rinsed three times in TBS. Rinsed sections were then incubated in Vectastain ABC Elite reagent for 1 h and developed using diaminobenzidine (Vector Laboratories). After dehydration all sections were mounted in Permount under a glass cover slip. Control sections were subjected to the identical staining procedure, except for the omission of the primary antibody.

### Protein extraction and western blot

Frozen rhesus macaque brain tissues were lysed using 1 × RIPA buffer (Sigma Aldrich), complemented with PIC (Protease Inhibitor Cocktail, Cat #11836153001, Roche) and Phosphatase Inhibitor Cocktail (Cat #11836153001, Roche). After protein quantification, samples were boiled for 10 min in SDS loading buffer (1:1 ratio). An aliquot (up to 50 μg) of the resulting sample was run on an SDS–PAGE gel and then transferred to a Hybond-PVDF membrane (Amersham Pharmacia). The membrane was blocked in 5% milk for 1 h at room temperature and incubated overnight with the appropriate primary antibody re-suspended in 5% milk in TBST at 4 °C. Following three-time washes using TBST, the membrane was incubated for 2 h at room temperature with the appropriate secondary antibody re-suspended in 5% milk in TBST at room temperature, followed by four washes using TBST. Signal ECL (Pierce) amplification was detected by Tanon 5200 Multi chemiluminescent image system (Tanon, Shanghai, China). Signal intensity was quantified with Image J (National Institute of Health).

### BASEscope assays for circRNA detection

BASEscope assays were performed using BaseScope™ Detection Reagent Kit-RED (#322900-USM, Advanced Cell Diagnostics (ACD) Hayward, CA) in accord with the manufacturer′s protocol. CircCACNA2D1 and circCACNA1E junction site-targeting or -nontargeting label probes conjugated to HRP were ordered from ACD. Briefly, 10 μm cryostat macaque brain sections were pretreated with Hydrogen Peroxide followed by performing target retrieval using an Oster^®^ Steamer. Dried slides were placed on the slide rack, than incubated with RNAscope^®^ Protease III at 40 °C for 30 min in the HybEZ™ system (ACD). Next, the slides were incubated at 40 °C in order to hybridize probes (circCACNA2D1 and circCACNA1E) for 2 h in HybEZ™ system. The slides were then performed signal amplification with the following steps: AMP 0 for 30 min; AMP 1 for 15 min; AMP 2 for 30 min; AMP 3 for 30 min; AMP 4 for 15 min; AMP 5 for 30 min; AMP 6 for 15 min. After each step, slides were washed with wash buffer three times at room temperature. Chromogenic detection was performed using BaseScope™ Fast RED followed by counterstaining with hematoxylin (American MasterTech Scientific, Lodi, CA). All images were collected using a Zeiss Olympus IX‐81 microscope with either a 40× or 100× objective running Metamorph. For image analysis, regions of interest (50 × 50 μm) were manually drawn, and after background subtraction, BASEScope intensity was normalized to the control. Images were thresholded in Image J and, using the Image Calculator tool, a third image was generated showing those pixels which were positive in all input channels. Using the Particle Analysis tool, the size and number of the thresholded clusters were analyzed. Microsoft Excel was used to calculate the fraction of positive clusters. GraphPad Prism was used to perform ANOVAs and *t*-tests and to visualize bar charts. Error bars represent SEM. The target genes, probed regions, and sequences of target probes are listed in Supplementary Table S2.

### DIG-labeled anti-sense RNA probes synthesis by in vitro transcription

PCR primers were designed using standard primer designing tools (Primer Premier 5.0) to amplify 100-300 nt fragment corresponding to linear and circular RNA sequences or 100-150 nt fragment corresponding to head-to-tail junction (short circRNA specific probes and not overlapping with linear RNA sequence). T7 promoter sequence was added to the reverse primer to obtain an antisense probe in vitro transcription reaction. In vitro transcription was performed using T7 RNA polymerase (Roche) with DIG-RNA labeling mix (Roche) according to manufacturer’s instruction. DNA templates were removed by DNAs I digestion and RNA probes purified by phenol chloroform extraction and subsequent precipitation. Probes were used at 50 ng/ml (Northern blot) final concentration. The primer sequences of probes were seen in Supplementary Table S4.

### Northern Blots for circRNA detection

Total RNA (10 μg for 10- and 20-year-old rhesus macaque brain samples, 2 μg for fetal macaque hippocampal primary neurons) was denatured using NorthernMax^®^-Gly sample loading dye (Ambion) and resolved on 1.2% agarose gel in MOPS buffer. The gel was soaked in 1 × TBE for 20 min and transferred to a Hybond-N + membrane (GE Healthcare) for 1 h (15 V) using a semi-dry blotting system (Bio-Rad). Membranes were dried and UV-crosslinked with 150 mJ/ cm^2^ at 254 nm. Pre-hybridization was done at 68 °C for 1 h, and using DIG Northern Blot Starter KIT (Roche) DIG-labeled in vitro transcribed circCACNA2D1, circCACNA1E, CDRA1s, and circMbl junction site-targeting probes were hybridized overnight. The membranes were washed three times in 2 × SSC, 0.1% SDS at 68 °C for 30 min, followed by three 30 min washes in 0.2 × SSC, 0.1% SDS at 68 °C. The immunodetection was performed with anti-DIG AP-conjugated antibodies. Immunoreactive bands were visualized using CDP star reagent (Roche) and a LAS-4000 detection system (GE Healthcare).

### Statistical analysis

Principal component analysis (PCA) was used to analysis the expression pattern of circRNAs (Fig. 3c). Permutation test was performed by R software. Two-sided paired *t*-test (Figs. 3a, 4a and 5a) were performed to calculate the differently expressed circRNAs. Hypergeometric distribution test was used to define the enrichment of each GO term and KEGG pathway (Fig. 3i, 6f, Supplementary Figs. S2b, c, and S4f-h,). Hierarchical clustering was performed to cluster the circRNAs by normalized value using Cluster3.0. Java TreeView softwares^60^. The statistical significance of the in situ hybridization data was tested using either an unpaired *t* test or Mann–Whitney *U* test since the normality of the distribution was pretested using Lilliefors test. No statistical methods were used to predetermine sample sizes, but our sample sizes are similar to those generally employed in the field. Data collection and analysis were not performed blind to the conditions of the experiments and no randomization of data was performed.

### Data accession numbers

The circRNA-seq data have been deposited in GEO and are accessible through accession number GSE94027.

The poly(A)-enriched RNA-seq data has been deposited in GEO and are accessible through accession numbers GSE85377^40^.

## Electronic supplementary material

Supplementray Information

Supplementary Table S3

Supplementary Table S4

## Electronic supplementary material

**Supplementary Information** accompanies the paper at (10.1038/s41421-018-0050-1).
