# Supplementary material for: Annotation and functional clustering of circRNA expression in rhesus macaque brain during aging
Source: Cell Discov. 2018 Sep 18;4:48. doi: 10.1038/s41421-018-0050-1 (PMC6141548; doi:10.1038/s41421-018-0050-1)
Supplement: Supplementary file 1 — Supplementray Information [file 41421_2018_50_MOESM1_ESM.pdf]

Supplementary information

Supplementary Table

Table S1. Profile of the Samples sequenced and the circRNA detection from our study

Samples	Area	Sex	Age	Raw	Clean	Clean%	Mapping%	circRNA (CIRI2)	circRNA (find_circ)
PFC_10F	Prefrontal cortex	Female	10	37085768	34189946	92.19%	25665366(75.07%)	7896	4195
PFC_10M		Male	10	44552154	39992978	89.77%	25741979(64.37%)	10052	5555
PFC_20F		Female	20	34952908	34571038	98.91%	31762744(91.88%)	4126	2049
PFC_20M		Male	20	42581844	37922024	89.06%	24608484(64.89%)	8527	4845
PCC _10F	Posterior Cingulate Cortex	Female	10	112492978	92227320	81.98%	37572158(40.74%)	6587	3981
PCC_10M		Male	10	107822534	87529702	81.18%	34803738(39.76%)	3563	2090
PCC_20F		Female	20	47565028	46386300	97.52%	40938814(88.26%)	6326	3047
PCC_20M		Male	20	57746240	49859186	86.34%	21785985(43.70%)	4114	2502
TC_10F	Temporal Cortex	Female	10	96553794	82305672	85.24%	36626765(44.50%)	5309	3220
TC_10M		Male	10	60218370	51122922	84.90%	19630873(38.40%)	4594	3192
TC_20F		Female	20	79963220	72137230	90.21%	35958355(49.85%)	5217	3159
TC_20M		Male	20	91568052	78721862	85.97%	36248094(46.05%)	6163	3802
PC_10F	Parietal Cortex	Female	10	38606352	34591020	89.60%	24291466(70.22%)	9004	4822
PC_10M		Male	10	42476014	41209828	97.02%	37508821(91.02%)	5045	2572
PC_20F		Female	20	82847958	70822324	85.48%	35217897(49.73%)	8215	4522
PC_20M		Male	20	48308152	42076788	87.10%	24916034(59.22%)	7241	4107
OC_10F	Occipital Cortex	Female	10	99500104	85869742	86.30%	46496978(54.15%)	9028	5630
OC_10M		Male	10	91434186	77220176	84.45%	41402570(53.62%)	6013	3883
OC_20F		Female	20	103084226	87288996	84.68%	42193607(48.34%)	7956	5234
OC_20M		Male	20	104682616	90826774	86.76%	53787731(59.22%)	10927	6934
CA1_10F	Cornu Ammonis 1	Female	10	39913286	34332926	86.02%	17019228(49.57%)	5631	3879
CA1_10M		Male	10	70758106	63320964	89.49%	29855096(47.15%)	4210	2749
CA1_20F		Female	20	42721806	37515580	87.81%	18756531(50.00%)	3014	1880
CA1_20M		Male	20	50313028	49344116	98.07%	45559536(92.33%)	6703	3113
DG_10F	Dentate Gyms	Female	10	66172838	52426962	79.23%	21833545(41.65%)	2968	1845
DG_10M		Male	10	79924358	62003594	77.58%	24768273(39.95%)	3782	2475
DG_20F		Female	20	70008154	56593170	80.84%	26175752(46.25%)	3713	2427
DG_20M		Male	20	45685786	44625182	97.68%	38241131(85.69%)	4890	2552
CB_10F	Cerebellar cortex	Female	10	81175942	65832248	81.10%	22205951(33.73%)	2284	1671
CB_10M		Male	10	61975696	51481292	83.07%	22151122(43.03%)	9200	6166

CB_20F		Female	20	70203504	64418050	91.76%	31674702(49.17%)	8161	5422
CB_20M		Male	20	60975246	50307188	82.50%	22148062(44.03%)	7243	5115

Table S2. Sequences of siRNAs and BASEScope assay probes targeting circRNAs

circRNA (rhesus macaque)	Sequences of siRNAs and BASEScope assay probes	
Cacna2d1	junction site (red)	5'-agaucuaaagcccuggugggaucaaaucaugggugga-3'
	siRNA-control 1	3'-uggugcuaguuuaguaccc-5'
	siRNA 1	3'-accaccuaguuuaguaccc-5'
	siRNA-control 2	3'- gauuuxgggaccacgauca-5'
	siRNA 2	3'- gauuuxgggaccaccuagu-5'
	BaseScope™ Target Probe	5'-tgatttgatccaccagggctttagatctgttgcttagaagtttctca-3' (BA-Mmu-CACNA2D1-circRNA-Junc 1zz targeting 63-109 of the provided sequence >3:134908228|134923364_+)
Cacna1e	junction site (red)	5'-ggaccaccgugcuguacaagcauccuggccacugcg-3'
	siRNA-control 1	3'-guacaucguaggaccggug-5'
	siRNA 1	3'-cauguucguaggaccggug-5'
	siRNA-control 2	3'-guggcacgacauguagcau-5'
	siRNA 2	3'-guggcacgacauguucgua-5'
	BaseScope™ Target Probe	5'-gccaggatgcttgtacagcacggtggtccacccttcca-3'(BA-Mmu-CACNA1E-circRNA-Junc 1zz targeting 193-230 of the provided sequence >1:189113029|189116190_-)

Supplementary Figures

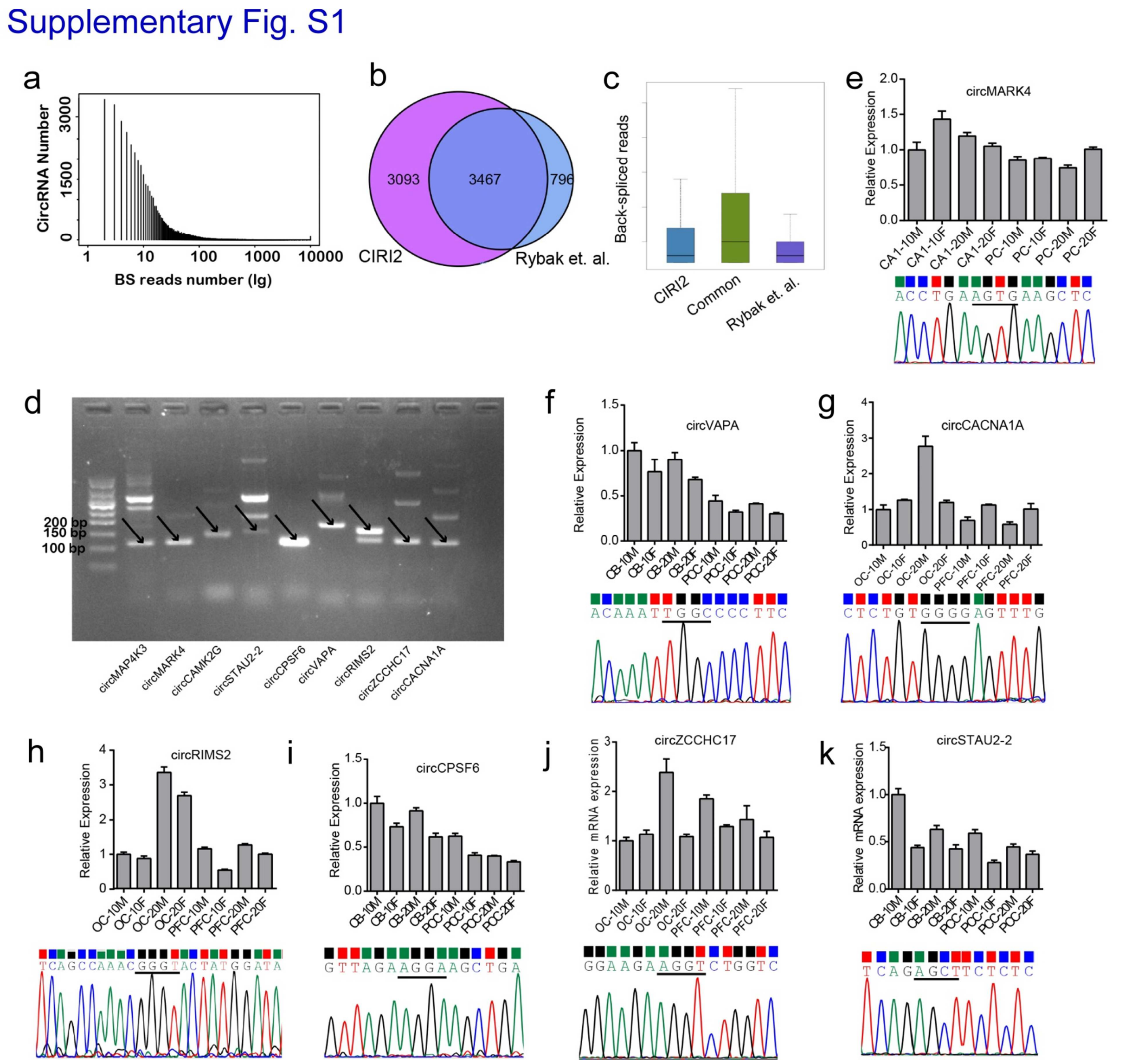

Supplementary Fig. S1

Annotation and characteristics of circRNA expression in rhesus macaque brain

(**a**) Bar plot showing the distribution of circRNA number by different back splicing reads count. Most circRNAs were low expressed with no more than 10 back splicing reads.

(**b**) Venn diagram showing the overlap between the published circRNAs and the predicted circRNAs by CIRI2.

(**c**) Box plot of the circRNA expression level by different groups: published specific, CIRI2 specific and theirs overlapped. The expression level of specific circRNAs was significant lower than that of overlapped circRNAs.

(**d**) Gels presentation of the circRNAs that were validated by Sanger sequencing. The arrows represent the location of back-splicing fragments. Nine circRNAs were selected as presentation.

(**e**) CIRI2 specifically detected circMARK4 was validated RT-qPCR and Sanger sequencing for sequences around the back splicing sites. The bases above the black line indicate the back splicing junction.

(**f-k**) Co-detected circRNAs by CRIR2 and find_circ were validated by RT-qPCR and Sanger sequencing. The presentation format was the same with (**e**).

Supplementary Fig. S2

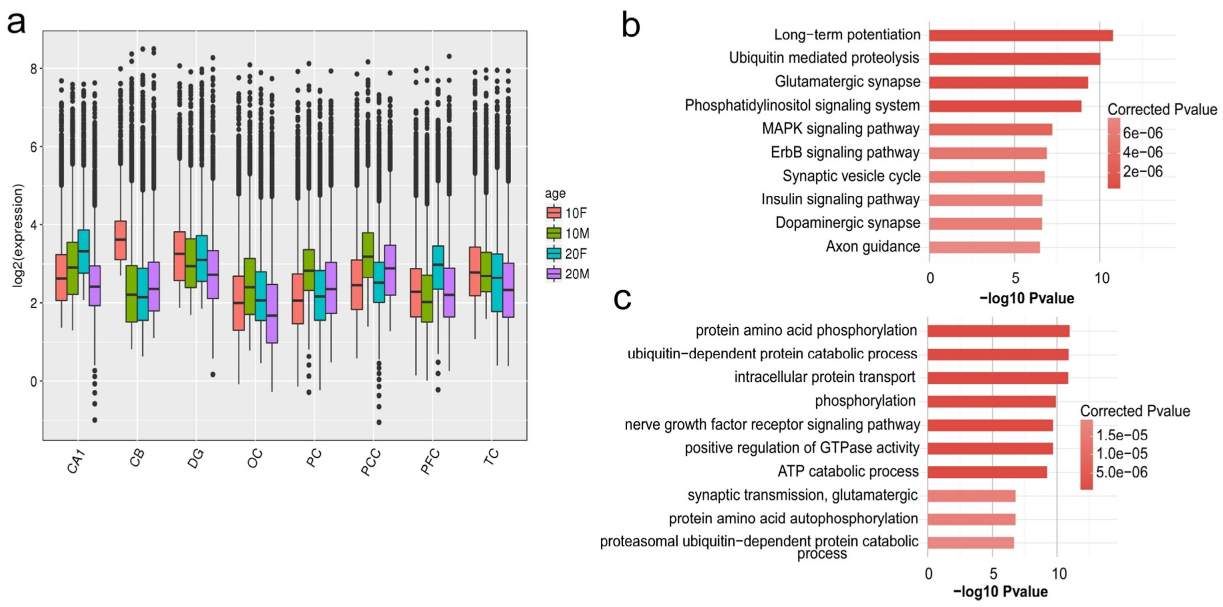

Supplementary Fig. S2

Patterns and functional analysis of circRNA expression in rhesus macaque brain

(**a**) Box plot showing the expression pattern of circRNAs from all the sex- and age-matched 32-paired samples.

(**b**) Bar plot showed the top 10 enriched KEGG pathways for the circRNA host genes.

(**c**) Bar plot showed the top 10 enriched biological process terms from GO database for the circRNA host genes.

Supplementary Fig. S3

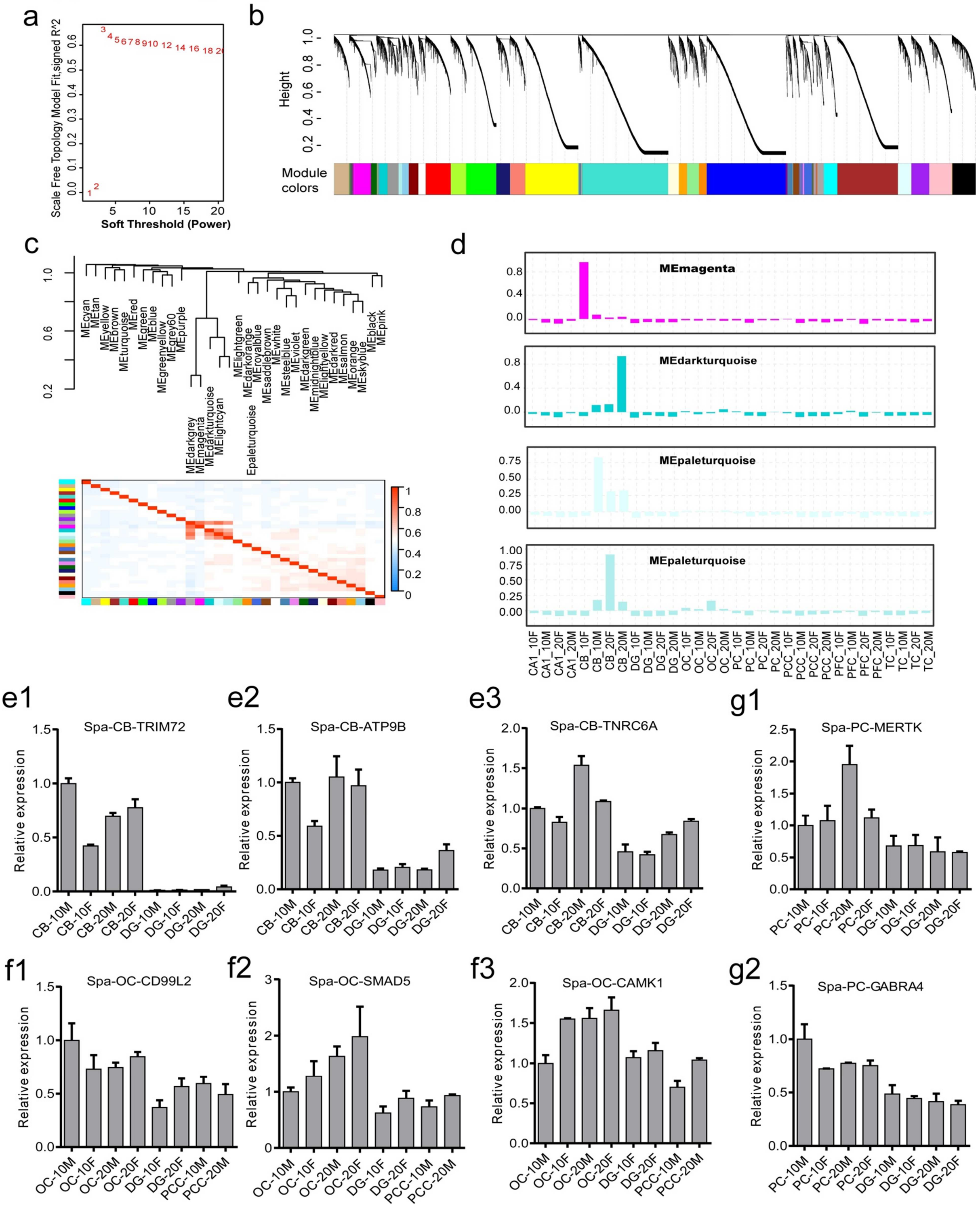

Supplementary Fig. S3

Analysis and characteristics of spatial-related circRNAs in rhesus macaque brain

(**a**) Power estimation was calculated according to the scale free topology model. Cubic power was chosen as the soft threshold.

(**b**) Clustering dendrogram showing the distinct circRNA expression pattern by WGCNA analysis. Bottom color bar represents the modules for each color.

(**c**) Eigengene dendrogram and heatmap for all WGCNA module eigengene value. The heatmap represents correlation coefficients between any module pairs.

(**d**) Bar plot of the eigengene values for the selected WGCNA modules. All the four modules (magenta, darkturquoise, paleturquoise, and paleturquoise) showed the CB enriched expression pattern compared with other modules.

(**e1-3**) Bar plot presentation for the RT-qPCR results showed three CB enriched circRNAs compared with other brain areas, including circTRIM72 (left), circATP9B (middle), circTNRC6A (right).

(**f1-3**) Bar plot presentation for the RT-qPCR results showed three OC enriched circRNAs compared with other brain areas, including circCD99L2 (left), circSMAD5 (middle), circCAMK1 (right).

(**g1-2**) Bar plot presentation for the RT-qPCR results showed two PC enriched circRNAs compared with other brain areas, including circMERTK (top panel), and circGABRA4 (bottom panel).

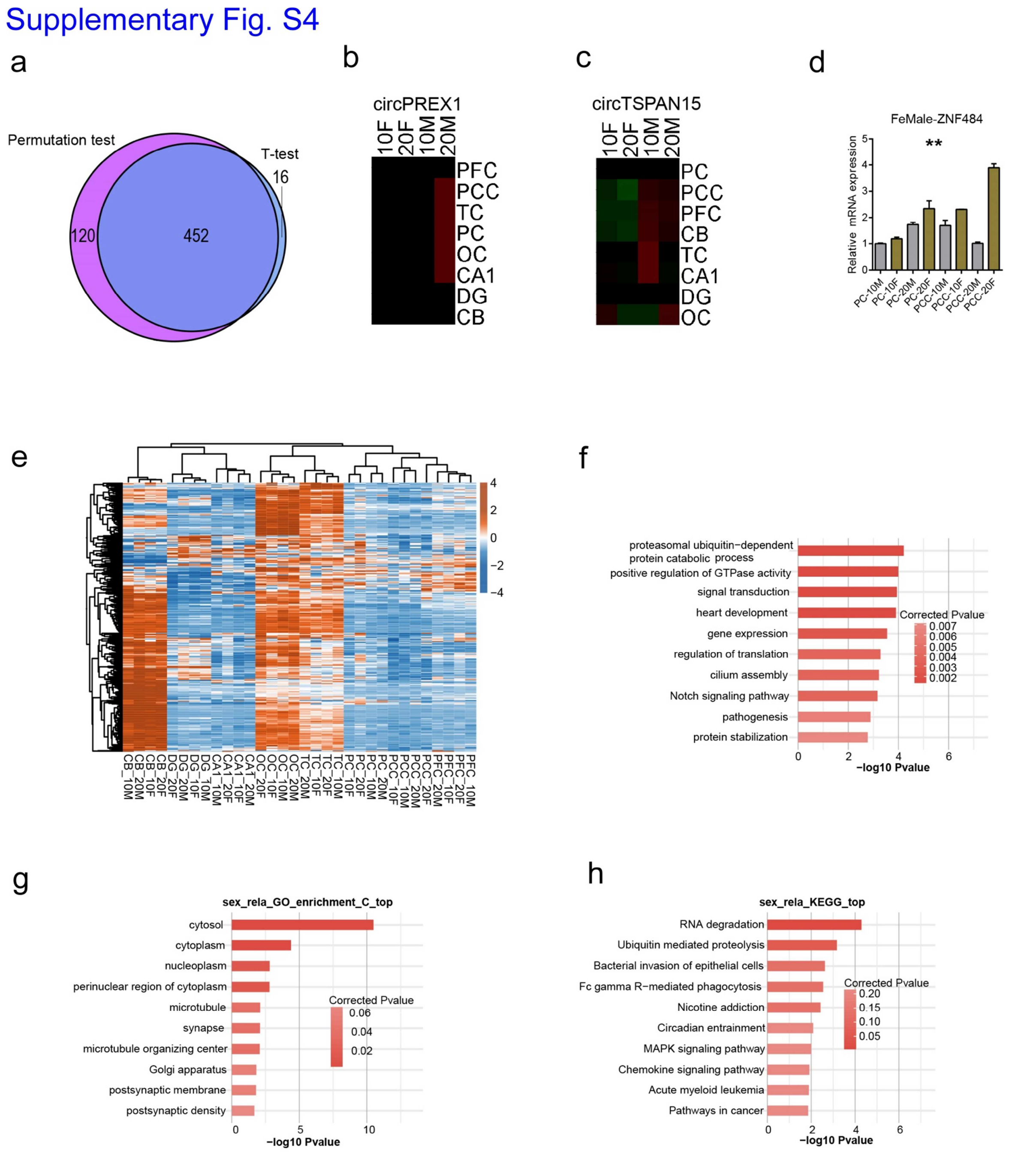

Supplementary Fig. S4

Identification and characteristics of sex-biased circRNAs in rhesus macaque brain

(**a**) Venn diagram showing the overlapping result of sex-biased circRNAs detected by permutation test and *t*-test.

(**b**) Heatmap of sex-specific circRNAs (circPREX1) showed male-specific feature, especially for 20y male sample.

(**c**) Heatmap of sex-specific circRNAs (circTSPAN15) showed male-specific feature, especially for 10y male sample.

(d) Heatmap of the host linear gene expression level for sex-bias circRNAs. Tissue bias expression pattern was dominant for these genes.

(e) Bar plot presentation for the RT-qPCR validation of female-biased circZNF484. Two brain regions were selected for validation.

(f) Bar plot of top 10 KEGG pathways for the host genes of sex-specific circRNAs.

(**g-h**) Bar plot of functional enrichment GO terms and KEGG pathways in sex-related circRNAs. Each bar represents cellular component, molecular function, biological process of GO terms and KEGG pathways, respectively.

Supplementary Fig. S5

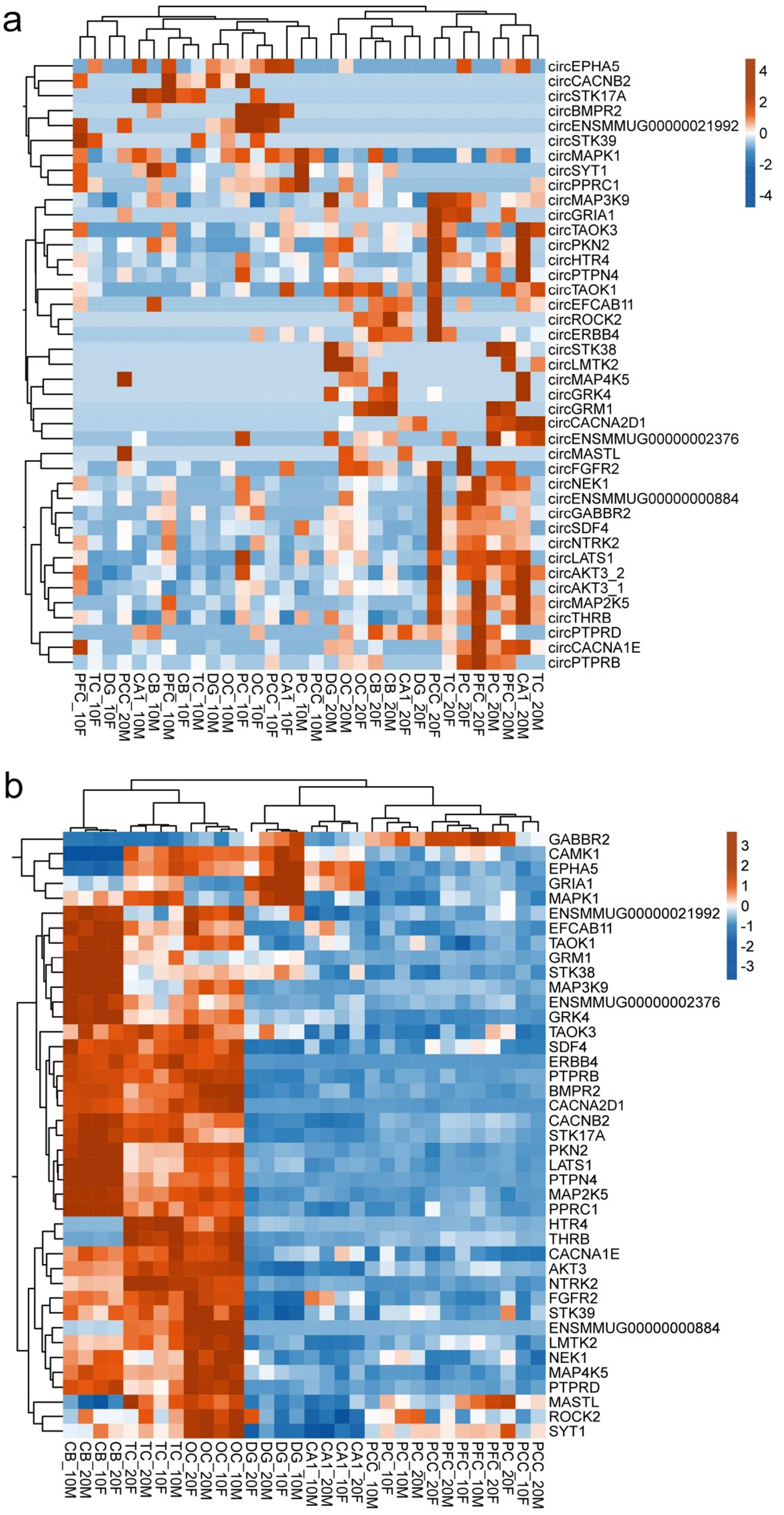

Supplementary Fig. S5

Negative correlation between age-related circRNA and host mRNA expression over aging states

(**a**) Heatmap of age-related circRNA expression showing the increased levels in several circRNA clusters in PFC, PCC, TC and CA1 at which typically loss of calcium homeostasis and homeostatic synaptic plasticity during brain ageing.

(**b**) Heatmap of levels of age-related circRNA host mRNA expression showing the decreased levels in PFC, PCC, TC and CA1.

Supplementary Fig. S6

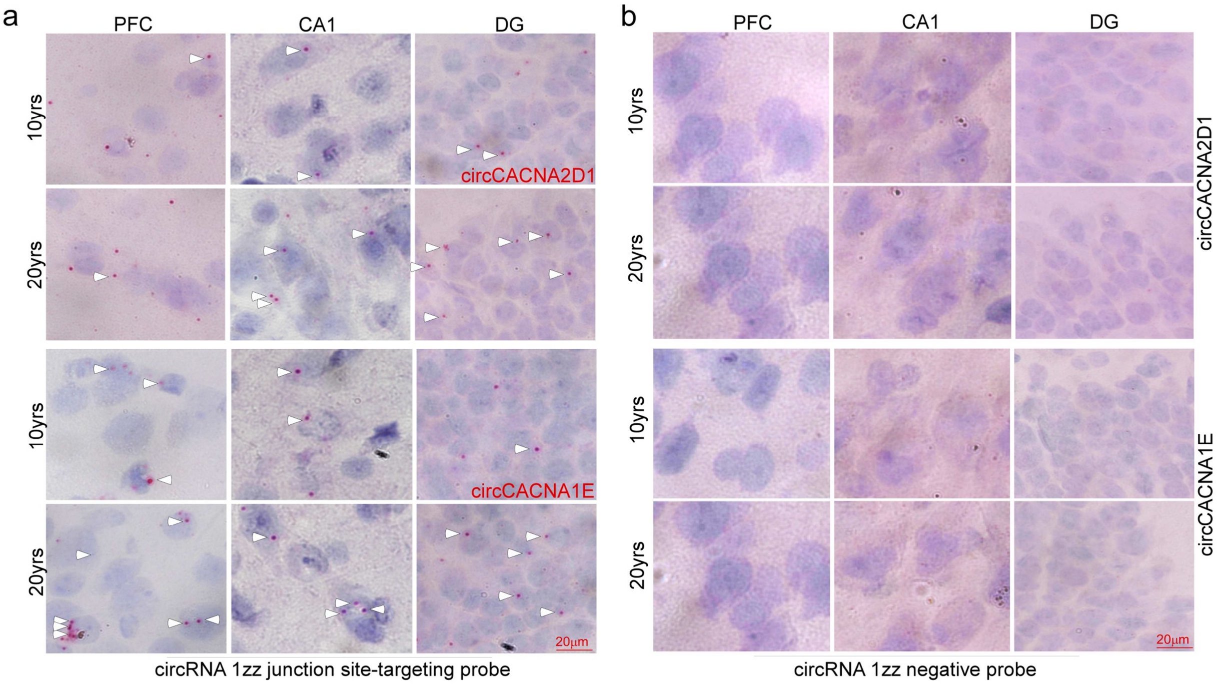

Supplementary Fig. S6

Increased nuclear distribution of circCACNA2D1 and circCACNA1E in neurons of aged macaque brain

(**a**) PFC and hippocampal ten-micron cryostat sections of 10- and 20-year-old sex-matched macaques were performed *BASEscope* with 1zz junction site-targeting probes against circCACNA2D1 and circCACNA1E, respectively (Red dots with nuclei counterstaining by

hematoxylin). Arrows represent nuclear localization of circRNAs. Scale bar, 20 μm. The images are representative of replicates of three independent experiments.

(**b**) PFC and hippocampal ten-micron cryostat sections of 10- and 20-year-old sex-matched macaques were performed *BASEscope* with the junction site-nontargeting probes (negative control), respectively (nuclei counterstaining by hematoxylin). Scale bar, 20 μm. The images are representative of replicates of three independent experiments.

Supplementary Fig. S7

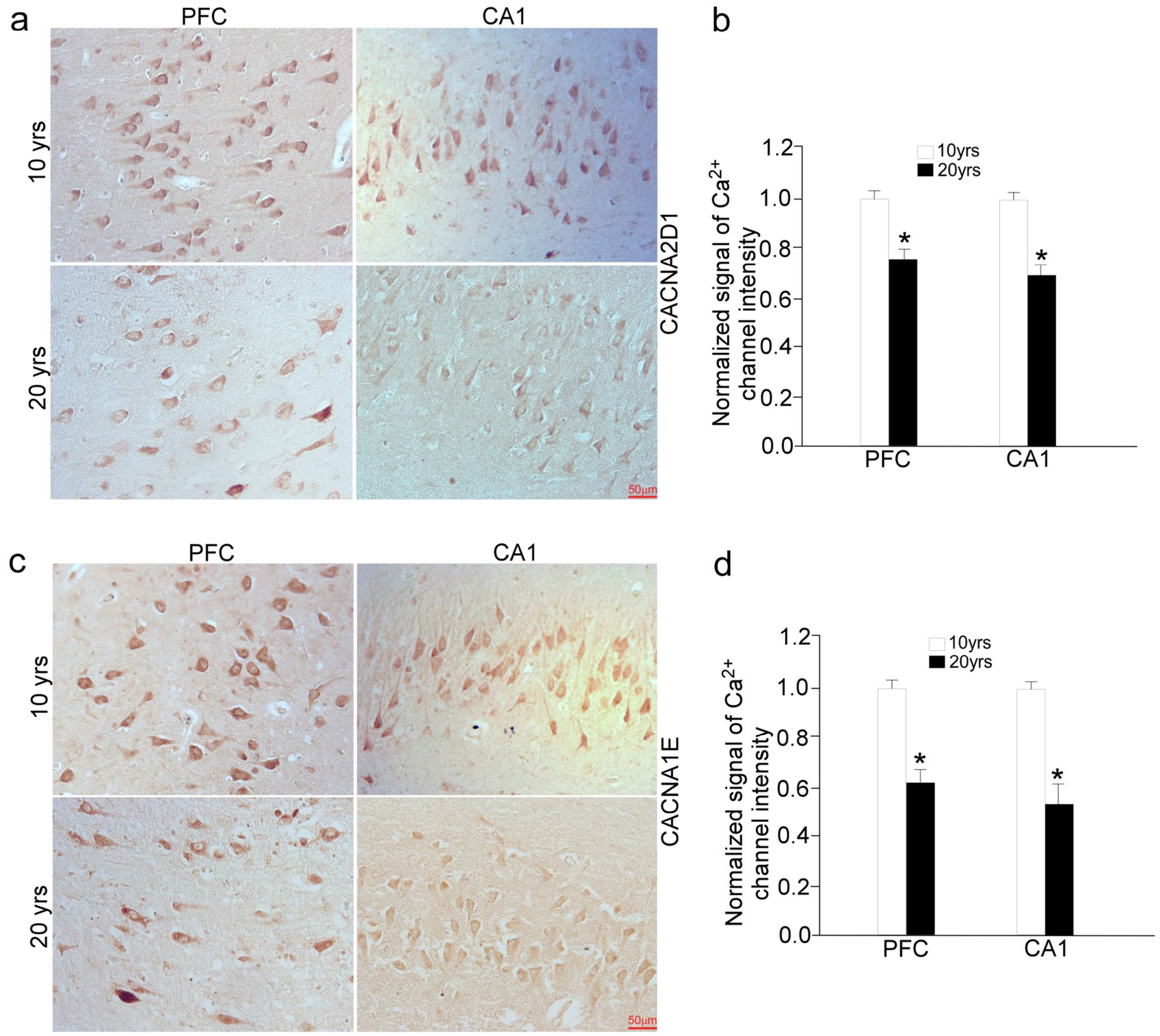

Supplementary Fig. S7

Age-related decreases in CACNA2D1 and CACNA1E protein levels **found in the aged brain of macaque**

(**a**) Ten-micron cryostat sections of 10- and 20-year-old sex-matched macaque PFC and hippocampus were immunostained with L-type voltage-dependent calcium channels CACNA2D1 antibody using HRP immunocytochemistry (brown). Scale bar, 50 µm.

(**b**) Relative intensities of CACNA2D1 immunostaining illustrated in panel A) were quantified by use of image J software. Data are present as mean ± s.e.m. (*n*=28-37 cells per group). Each

bar represents the average of three independent experiments; error bars denote s.e.m. (*, $p < 0.05$, unpaired *t* test).

(**c**) Ten-micron cryostat sections of 10- and 20-year-old sex-matched macaque PFC and hippocampus were immunostained with R-type voltage-dependent calcium channels CACNA1E antibody using HRP immunocytochemistry (brown). Scale bar, 50 μm.

(**d**) Relative intensities of glutamate receptors immunostaining illustrated in panel **c**) were quantified by use of image J software. Data are present as mean ± s.e.m. (*n*=31-43 cells per group). Each bar represents the average of three independent experiments; error bars denote s.e.m. (*, $p < 0.05$, unpaired *t* test).

Supplementary Fig. S8

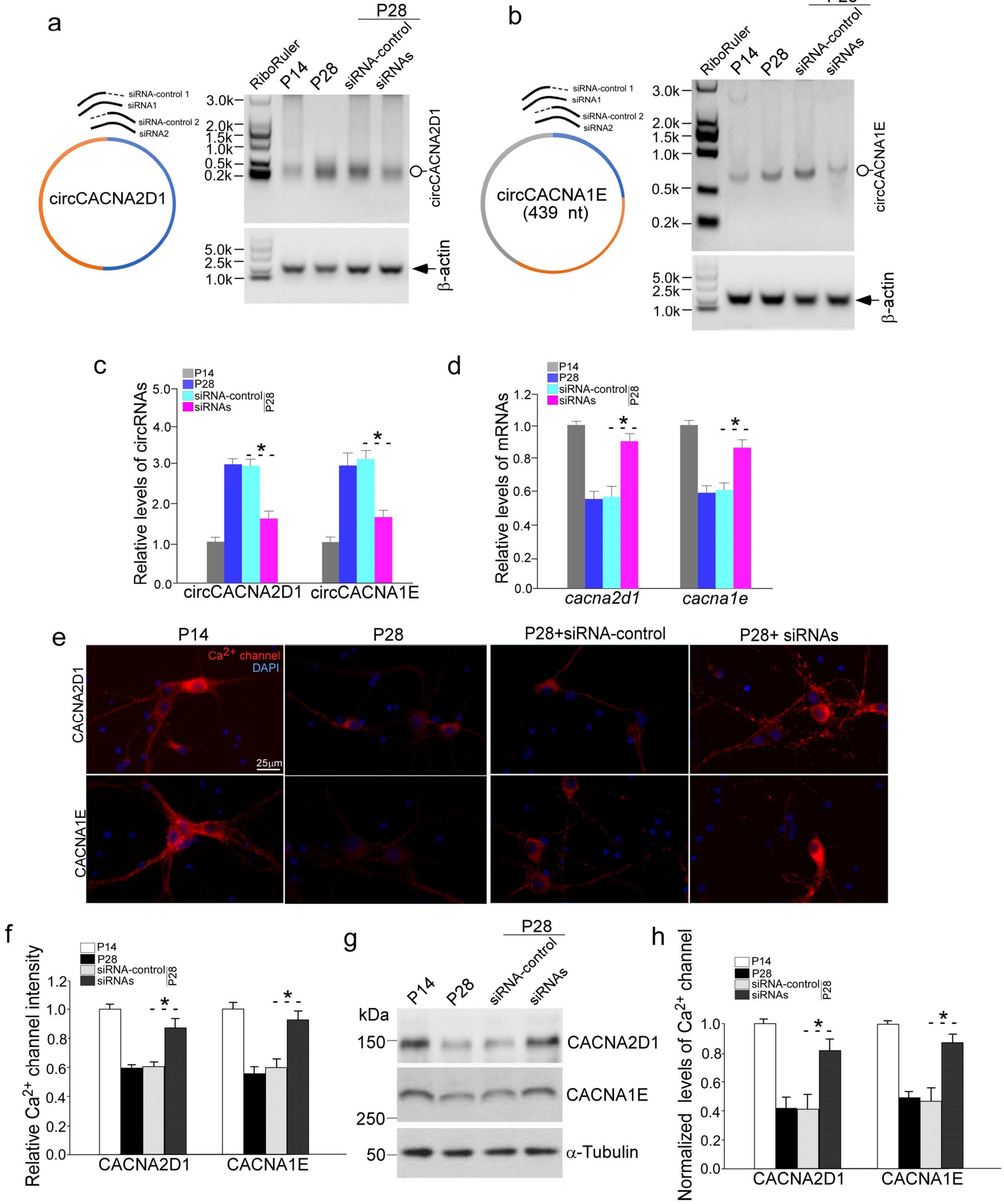

Supplementary Fig. S8

CircCACNA2D1 and circCACNA1E negatively correlate to host mRNA expression over aging states

(**a**-**b**) Northern blots of *cacna2d1* and *cacna1e* circular transcripts. Total RNAs were extracted from P14, and P28 fetal macaque hippocampal neurons infected at P7 with the junction site-targeting siRNAs against circCACNA2D1 and circCACNA1E*,* or the mismatched junction site-targeting, and digested with RNase R. Northern blots were performed on the basis of hybridization with the specific digoxin-labeled junction site-targeting probes showing that that circCACNA2D1 and circCACNA1E were efficiently knocked down with siRNAs in fetal macaque hippocampal neurons. K denotes 1,000 nt. The blots represent one of three independent experiments. The siRNAs targeted circCACNA2D1 and circCACNA1E at junction sequences. The blue and yellow (circCACNA2D1), or the blue and gray (circCACNA1E) sequences below the histograms show the corresponding exon sequences forming the junction; mismatched sequences in the corresponding control siRNA are shown in black.

(**c**-**d**) Total RNAs were extracted from P14, P28 fetal macaque hippocampal neurons infected at P7 with the junction site-targeting siRNAs against circCACNA2D1 and circCACNA1E ,or the mismatched junction site-targeting siRNA-control, and performed qPCR for circCACNA2D1, circCACNA1E and their host mRNA expression. Data are presented as mean ± SD, n=3 technical replicates. *, $p< 0.01$, unpaired *t* test.

(**e**) Fetal macaque hippocampal neurons were infected at P7 with either the junction site-targeting siRNAs against circCACNA2D1 and circCACNA1E or the mismatched junction site-targeting siRNA-control. On P14 or P28, neurons were fixed and immunostained with CACNA2D1 and CACNA1E antibodies. Scale bar =25 μm.

(**f**) Relative intensities of Ca^{2+} channels immunostaining illustrated in panel **e**) were quantified by use of image J software. Data are present as mean ± s.e.m. (*n*=27-38 cells per group). Each bar represents the average of three independent experiments; error bars denote s.e.m. *, $p< 0.05$, unpaired *t* test.

(g) Protein extracts from P14, P28 fetal macaque hippocampal neurons infected at P7 with the junction site-targeting siRNAs against circCACNA2D1 and circCACNA1E, or the mismatched junction site-targeting siRNA-control, were immunoblotted with CACNA2D1 and CACNA1E antibodies. α-Tubulin was loading control. The blots represent one of three independent experiments.

(**h**) Normalized protein levels of Ca^{2+} channel CACNA2D1 and CACNA1E illustrated in panel **g**) were quantified by use of image J software. Data are present as mean ± s.e.m. Each bar represents the average of three independent experiments; error bars denote s.e.m. *, $p< 0.05$, unpaired *t* test.

Supplementary Materials and Methods

Animals and Samples collection

Frozen postmortem brain tissue samples from rhesus macaque were provided by Kunming Primate Research Center of the Chinese Academy of Sciences (KPRC). Brain regions were systematically collected from well-characterized rhesus monkeys born and raised at the KPRC in outdoor, 6-acre enclosures that provide a naturalistic setting and normal social environment. For circRNA-seq analysis, two male and two female rhesus macaque brain specimens at each of stages representing adult (10-year-old) and old (20-year-old) were profiled. Extensive health, family lineage and dominance information were maintained on all animals.

According to a widely used macaque brain atlas and brain maps (http: //www.Brainmaps.org), tissues spanning eight anatomically distinct regions were selected and collected from each specimen. The detailed information was described as below: the prefrontal cortex was sampled at the main sulci, the posterior cingulate cortex was sampled at the Brodmann's area 23, the temporal cortex at the superior temporal gyrus, the parietal cortex at the middle sylvian fissure, the occipital cortex at the V1, and the cerebellar cortex was sampled at the cauda cerebellum. The hippocampus (including CA1 and dentate gyrus) was also sampled. All the collected samples were washed with RNA later solution (AM7021, Ambion, USA) and put in freezing tubes to store at liquid nitrogen temperature.

All animal procedures were in strict accordance with the guidelines for the National Care and Use of Animals approved by the National Animal Research Authority (P.R. China) and the Institutional Animal Care and Use Committee (IACUC) of the Kunming Institute of Zoology of Chinese Academy of Sciences. The nonhuman primate cares and experimental protocols were approved by the Ethics Committee of Kunming Institute of Zoology and the Kunming Primate Research Center, Chinese Academy of Sciences (AAALAC accredited), and the methods were carried out in accordance with the approved guidelines.

CircRNA library preparation

Before the preparation of the circRNA-seq libraries, the total RNA samples for two males were mixed and those for females were mixed as well to eliminate the individual biases. Samples were collected from eight different brain regions, and consequently a total of 32 mixed samples from

10-year male and female, and from 20-year male and female were subjected to library preparation. For each sample, 10µg of total RNA was used for cirRNA-seq library preparation. Total RNA was treated with RQ1 DNase (Promega) to remove genomic DNA. A large proportion of mRNA was captured by oligo dT, and remaining RNA was purified with Ampure XP Beads. Ribosomal RNA was depleted away by Ribo-Zero Magnetic Gold Kit (Human/Mouse/Rat, MRZG12324, Epicentre). Linear RNA (including residual rRNA, residual mRNA, and so on) was digested by RNase R.

CircRNA was iron fragmented at 95℃ followed by end repair and 5' adaptor ligation. The reverse transcription was performed with RT primer harboring 3' adaptor sequence and randomized hexamer. The cDNAs were purified and amplified, then PCR products corresponding to 300~500bp were selectively captured and quantified. Libraries were stored at -70 °C until used for sequencing. For high-throughput sequencing, the libraries were prepared following the manufacture's instructions and applied to Illumina NextSeq500 system with 150x2 paired-end type by ABlife Inc. (Wuhan, China).

circRNA Prediction and filtering

After reads were obtained from sequencing, Cutadapt (*1*) was used to trim adaptors (with 10% error rate) and low quality bases (less than 16). Reads that were less than 16nt in both ends were discarded. Before prediction, we used bwa(*2*) method to align reads to the *Rhesus Macaque* (*3*) reference genome (with parameters: -k 19 -T 19). After alignment, CIRI2 software (*4, 5*) was used to predict circRNAs in macaque brain samples. Candidate circRNAs with no less than two head-to-tail junction reads were preserved.

To test the precision and sensitivity of the CIRI2 software, we downloaded the raw data of the SH-SY5Y neuronal cells (*6*). We aligned the reads to the human genome (GRCH38 version) and predicted the circRNAs with our prediction pipeline with the same parameters. Then we converted the published circRNAs locus from GRCH37 to GRCH38 by liftOver (*7*). CircRNAs which splice sites were detected in +/- 5 nucleotide interval around the predicted circRNAs start or end were treated as overlap circRNAs. At the same time, we re-predicted circRNAs with our

data by utilizing find_circ (version 1.2) software with default parameters (*8*). Overlapping analysis was performed for the prediction results between CIRI2 and find_circ.

CircRNAs are constituted by two parts by CIRI2: back splicing junction part and non-junction part. After obtaining the circRNAs, reads number across the back splicing junctions was treated as the raw expression level of each circRNAs. After that, we filtered the circRNAs expressed only in sporadic samples with low abundance. Based on the back-splicing reads number of each circRNA, we set the following requirement as the filtering threshold:

1) No less than 3 back-splicing reads was required to define an expressed circRNA.

2) One brain area is defined as expressed only if no less than 2 samples satisfied the 1st requirement.

3) One circRNA is expressed only if on less than 2 areas were expressed.

4) If circRNAs can't meet the 3rd requirement, the circRNAs must be expressed in only one brain area and the total expressed samples must be on less than 6 (20% of total samples).

5) If circRNAs can't meet the 3rd and 4th requirements, the total back-splice reads number of this circRNA must be no less than 30.

To obtain a normalized expression between different samples, we used the DESeq normalization method (*9*) to obtain the normalized expression level for circRNAs by dividing a size factor.

Conservation analysis for macaque circRNAs

To analyze the homologous feature between macaque circRNAs and other mammalian species, we downloaded the human and mouse circRNAs sequence from the circBase (*10*). Then we aligned the circRNA sequence to the human and mouse circRNAs by blastn with E-value 1e-3. Meanwhile, we also adopted the conservation calculation method in Rybak-Wolf et al (*6*), and performed this analysis among three species: macaque, mouse, and human.

Characteristic analysis of circRNA expression

To find the characteristic of circRNA expression in macaque brain, we compared the circRNA expression pattern from multiple dimensions. First, we used WGCNA method (*11*) to classify circRNAs. Cubic power was chosen as the soft threshold to calculate block wise modules. The

output is the circRNA modules according to their expression pattern. For each circRNA module, eigengenes was chosen to represent the expression pattern.

Due to the low and dynamic characteristic of circRNAs compared with host mRNAs, we used unpaired *t*-test model to filtrate the spatial, ageing and sexual-related circRNAs as described previously(*12*). For spatial-related circRNAs filtration, we compared one brain area with other areas by *t*-test and repeated this according the eight brain areas. For ageing-related circRNAs filtration, we compared 10-year-old samples with 20-year-old samples. For sexual-related circRNAs filtration, we compared female samples with male samples. After comparison, we set *p*-value 0.05 as the threshold for filtration.

Analysis of the relationships between circRNAs and host mRNAs

To explore the expression relationship of circRNAs and their host mRNAs, we used the poly(A) selected RNA-seq data from our another study (*13*). The biological samples of the two studies were the same and total RNAs were extracted from the brain tissue parallelly. So we could compare the expression level between circRNAs and host mature mRNAs. Based on the expression of each circRNA and host mRNA, Pearson correlation coefficient (PCC) and *p*-value were obtained for each mRNA-circRNA pair. Then we filter the result by a given threshold, absolute PCC no less than 0.3 and *p*-value no more than 0.1. Besides the positive correlation pairs, negative pairs with correlation coefficient less than 0 were also included.

RNA extraction and reverse transcription PCR

RNA was using PureLink micro-to-midi total RNA purification system (Invitrogen). RNA quantity and quality were evaluated by Nano Drop ND-1000 spectrophotometer (Nano Drop Thermo, Wilmington, DE) and RNA integrity was assessed by agarose gel electrophoresis. Specific divergent primers were designed to amplify the circular and linear Rhesus macaque transcripts. Semi-quantitative RT-PCR was performed with Superscript III one-step RT-PCR system with platinum Taq High Fidelity (Invitrogen). For quantitative real-time PCR, cDNAs were prepared by using oligonucleotide (dT), random primers, and a Thermo Reverse Transcription kit (Signal way Biotechnology). qPCR was performed with SYBR Green I Master

(Roche, 04707516001) on Light Cycler 480 II. qPCR was performed in 10 μl reaction volume, including 2 μl of cDNA, 5 μl 2× Master Mix, 0.5 μl of Forward Primer (10 μM), 0.5 μl of Reverse Primer (10 μM) and 2 μl of double distilled water. The reaction was set at 95 °C for 10 min for pre-denaturation, then at 95 °C for 10 s and at 60 °C for 60 s repeating 40 cycles. *GAPDH* was used as a reference. Both target and reference were amplified in triplicate wells. And the relative level of each circular and linear transcript was calculated using $2^{-\Delta\Delta Ct}$ method. All PCR primer sequences can be found in Supplementary Table S3.

Immunohistochemistry

For DAB/bright field staining, ten micron cryostat sections of macaque brain were pretreated in 0.3% hydrogen peroxide in methanol for 30 min to remove endogenous peroxidase activity, rinsed in Tris-buffered saline (TBS), and then treated with 0.1 M citrate buffer in a microwave at sufficient power to keep the solution at 100°C for 20 min. Sections were cooled in the same buffer at room temperature (RT) for 30 min and rinsed in TBS. Slides were incubated in 10% goat serum in PBS blocking solution for1 h at RT, after which primary antibodies CACNA2D1 (ab2864) and CACNA1E (ab63705) were applied to the sections that were then incubated at 4°C overnight. The sections were washed three times in TBS before applying the secondary antibody (Vector Laboratories). Secondary antibody was applied for 1 hour at RT. Afterwards, sections were rinsed three times in TBS. Rinsed sections were then incubated in Vectastain ABC Elite reagent for 1 h and developed using diaminobenzidine (Vector Laboratories). After dehydration all sections were mounted in Permount under a glass cover slip. Control sections were subjected to the identical staining procedure, except for the omission of the primary antibody.

Protein extraction and western blot

Frozen macaque brain tissues or cultured fetal hippocampal neurons were lysed using 1× RIPA buffer (Sigma Aldrich), complemented with PIC (Protease Inhibitor Cocktail, Cat #11836153001, Roche) and Phosphatase Inhibitor Cocktail (Cat #11836153001, Roche). After protein quantification, samples were boiled for 10 min in SDS loading buffer (1:1 ratio). An aliquot (up to 50 μg) of the resulting sample was run on a SDS–PAGE gel and then transferred to a Hybond-

PVDF membrane (Amersham Pharmacia). The membrane was blocked in 5% milk for 1 h at room temperature and incubated overnight with the appropriate primary antibody re-suspended in 5% milk in TBST at 4 °C. Following three time washes using TBST, the membrane was incubated for 2 h at room temperature with the appropriate secondary antibody re-suspended in 5% milk in TBST at room temperature, followed by 4 washes using TBST. Signal ECL (Pierce) amplification was detected by Tanon 5200 Multi chemiluminescent image system (Tanon, Shanghai, China). Signal intensity was quantified with Image J (National Institute of Health).

Fetal Rhesus macaque neural cell cultures

Fetal Rhesus macaque neuronal cultures were prepared with modification and maintained as described previously (*14*) . Briefly, fetal brains were obtained from second trimester fetal macaques (12～15 weeks) (Kunming Primate Research Center of the Chinese Academy of Sciences, KPRC) obtained via caesarian section. Animals were housed in accordance with standards of the nonhuman primate cares and experimental protocols were approved by the Ethics Committee of Kunming Institute of Zoology and the Kunming Primate Research Center, Chinese Academy of Sciences (AAALAC accredited), and the methods were carried out in accordance with the approved guidelines. The meninges were removed, and cortical tissue was mechanically dissociated using sterile scalpel blades and washed in sterile PBS. Cells were pelleted by centrifugation at 800 rpm for 10 min, and the pellet was subsequently enzymatically dissociated with 0.25% trypsin in the presence of DNase (50 μg/ml) at 37°C for 30 min. Cells were further mechanically dissociated through a 150-μm pore size nylon mesh filter and washed again in cold sterile PBS by centrifugation at 1500 rpm at 4°C for 10 min. The collected cell pellet was mechanically dissociated by trituration and further enzymatically dissociated with DNase (50 μg/ml) at RT for 10 min. After several further washes with cold sterile PBS, cells were seeded onto poly-D-lysine-coated glass coverslips or T75 flasks at a density of 1×10^6 cells/ml in DMEM/10% FCS. 5-Fluoro-2′-deoxyuridine (FDU; 10 μM), a uridine analogue that is toxic to dividing astrocytes, was added to cultures to inhibit over proliferation of glial cells on days 5, 8, and 12 in vitro. Cells were used for single- and double-label immunocytochemical techniques and calcium flux analyses after 1~4 weeks in culture.

siRNA knockdown in fetal macaque brain cultured neurons

Lentiviral siRNA preparation was prepared as described previously(*15*). piLenti™ -siRNA constructs including a set of circCacna2d1 and a set of circCacna1e were constructed using a standard protocol from Applied Biological Material Inc. The high lentiviral particle titers were prepared with 3rd Generation Packaging Mix in HEK 293T cells and purified by Ultra-Pure Lentivirus purification Kits (Applied Biological Material Inc. Vancouver). For knockdown assay, after 5-7 days in culture, fetal macaque hippocampal neurons were infected using a multiplicity of infection (moi) between 5 and 10 to provide an efficiency of infection above 70%, which piLenti™ –GFP was used a co-expressed reporter to monitor infection. Samples were collected at indicates time and different assays were performed. All sequences of siRNA against circRNAs can be found in Supplementary Table S2.

Immunofluorescence

Cultured fetal macaque hippocampal neurons were rinsed once with PBS and then fixed in buffered 4% paraformaldehyde in 0.1 M phosphate buffer for 30 min at room temperature followed by three rinses with PBS. Primary antibodies CACNA2D1 (ab2864) and CACNA1E (ab63705) concentrations used for cell culture were 1:200. All secondary antibodies were used at 1:1000. Cells were counterstained with 1 μg/ml DAPI. All immunofluorescence images were collected using a Zeiss Olympus IX-81 microscope with either a 40× or 100× objective running Metamorph. Microsoft Excel was used to calculate the fraction of positive clusters. GraphPad Prism was used to perform *t*-tests and to visualize bar charts. Error bars represent SEM.

BASEscope Assays for circRNA detection

BASEscope assays were performed using BaseScope™ Detection Reagent Kit-RED (#322900-USM, Advanced Cell Diagnostics (ACD) Hayward, CA) in accord with the manufacturer's protocol. CircCACNA2D1 and circCACNA1E junction site-targeting or -nontargeting label probes conjugated to HRP were ordered from ACD. Briefly, 10 μm cryostat macaque brain

sections were pretreated with Hydrogen Peroxide followed by performing target retrieval using an Oster® Steamer. Dried slides were placed on the slide rack, and incubated with RNAscope® Protease III at 40°C for 30 minutes in the HybEZ™ system (ACD). Next, the slides were incubated at 40°C in order to hybridize probes (circCACNA2D1 and circCACNA1E) for 2 HRS in HybEZ™ system. The slides were then performed signal amplification with the following steps: AMP 0 for 30 minutes; AMP 1 for 15 minutes; AMP 2 for 30 minutes; AMP 3 for 30 minutes; AMP 4 for 15 minutes; AMP 5 for 30 minutes; AMP 6 for 15 minutes. After each step, slides were washed with wash buffer three times at room temperature. Chromogenic detection was performed using BaseScope™ Fast RED followed by counterstaining with hematoxylin (American MasterTech Scientific, Lodi, CA). All images were collected using a Zeiss Olympus IX - 81 microscope with either a 40× or 100× objective running Metamorph. For image analysis, regions of interest (50 × 50 μm) were manually drawn, and after background subtraction, BASEScope intensity was normalized to the control. Images were thresholded in Image J and, using the Image Calculator tool, a third image was generated showing those pixels which were positive in all input channels. Using the Particle Analysis tool, the size and number of the thresholded clusters were analyzed. Microsoft Excel was used to calculate the fraction of positive clusters. GraphPad Prism was used to perform ANOVAs and *t*-tests and to visualize bar charts. Error bars represent SEM. The target genes, probed regions, and sequences of target probes are listed in Supplementary Table S4.

DIG-labeled anti-sense RNA probes synthesis by *in vitro* transcription

PCR primers were designed using standard primer designing tools (Primer Premier 5.0) to amplify 100-300 nt fragment corresponding to linear and circular RNA sequences or 100-150 nt fragment corresponding to head-to-tail junction (short circRNA specific probes and not overlapping with linear RNA sequence). T7 promoter sequence was added to the reverse primer to obtain an antisense probe *in vitro* transcription reaction. In vitro transcription was performed using T7 RNA polymerase (Roche) with DIG-RNA labeling mix (Roche) according to manufacturer's instruction. DNA templates were removed by DNAs I digestion and RNA probes purified by phenol chloroform extraction and subsequent precipitation. Probes were used at

50ng/ml (Northern blot) final concentration. The primer sequences of probes were seen in Supplementary Table S4.

Northern Blots for circRNA detection

Total RNA (10 μg for 10- and 20-year-old macaque brain samples, 2 μg for fetal macaque hippocampal primary neurons) was denatured using NorthernMax®-Gly sample loading dye (Ambion) and resolved on 1.2% agarose gel in MOPS buffer. The gel was soaked in 1×TBE for 20 min and transferred to a Hybond-N+ membrane (GE Healthcare) for 1 h (15 V) using a semi-dry blotting system (Bio-Rad). Membranes were dried and UV-crosslinked with 150 mJ/ cm^2 at 254 nm. Pre-hybridization was done at 68 °C for 1 h, and using DIG Northern Blot Starter KIT (Roche) DIG-labeled *in vitro* transcribed circCACNA2D1, circCACNA1E, CDRA1s, and circMbl junction site-targeting probes were hybridized overnight. The membranes were washed three times in 2× SSC, 0.1% SDS at 68 °C for 30 min, followed by three 30 min washes in 0.2× SSC, 0.1% SDS at 68 °C. The immunodetection was performed with anti-DIG AP-conjugated antibodies. Immunoreactive bands were visualized using CDP star reagent (Roche) and a LAS-4000 detection system (GE Healthcare).

Statistical analysis

Principal component analysis (PCA) was used to analysis the expression pattern of circRNAs (Supplementary Fig. S1d, S1f, Fig. 2b). Two-sided paired *t*-test (Fig. 2a, 3a, and 4a) were performed to calculate the differently expressed circRNAs. Hypergeometric distribution test was used to define the enrichment of each GO term and KEGG pathway (Fig. 2c, Supplementary Fig. S1g-h, S3c, and S4e). Hierarchical clustering was performed to cluster the circRNAs by TPM value using Cluster3.0. Java TreeView softwares (*16*). The statistical significance of the *in situ* hybridization data was tested using either an unpaired t test or Mann-Whitney *U* test since the normality of the distribution was pretested using Lilliefors test. No statistical methods were used to predetermine sample sizes, but our sample sizes are similar to those generally employed in the field. Data collection and analysis were not performed blind to the conditions of the experiments and no randomization of data was performed.

Data accession numbers

The circRNA-seq data has been deposited in GEO and are accessible through accession number GSE94027. Reviewers can view the original and processed data by the private link created by GEO:

https://www.ncbi.nlm.nih.gov/geo/query/acc.cgi?token=mfubicmonrkljcv&acc=GSE94027

The poly(A)-enriched RNA-seq data has been deposited in GEO and are accessible through accession numbers GSE85377(*13*).

References:

1. M. Martin, Cutadapt removes adapter sequences from high-throughput sequencing reads. *EMBnet.journal* **17**, 10–12 (2011).
2. H. Li, R. Durbin, Fast and accurate long-read alignment with Burrows-Wheeler transform. *Bioinformatics* **26**, 589-595 (2010).
3. S. Rhesus Macaque Genome *et al.*, Evolutionary and biomedical insights from the rhesus macaque genome. *Science* **316**, 222-234 (2007).
4. Y. Gao, J. Zhang, F. Zhao, Circular RNA identification based on multiple seed matching. *Briefings in Bioinformatics*, (2017).
5. S. Hoffmann *et al.*, A multi-split mapping algorithm for circular RNA, splicing, trans-splicing and fusion detection. *Genome biology* **15**, R34 (2014).
6. A. Rybak-Wolf *et al.*, Circular RNAs in the Mammalian Brain Are Highly Abundant, Conserved, and Dynamically Expressed. *Molecular cell* **58**, 870-885 (2015).
7. A. S. Hinrichs *et al.*, The UCSC Genome Browser Database: update 2006. *Nucleic acids research* **34**, D590-598 (2006).
8. S. Memczak *et al.*, Circular RNAs are a large class of animal RNAs with regulatory potency. *Nature* **495**, 333-338 (2013).
9. S. Anders, W. Huber, Differential expression analysis for sequence count data. *Genome biology* **11**, R106 (2010).
10. P. Glazar, P. Papavasileiou, N. Rajewsky, circBase: a database for circular RNAs. *Rna* **20**, 1666-1670 (2014).
11. P. Langfelder, S. Horvath, WGCNA: an R package for weighted correlation network analysis. *BMC bioinformatics* **9**, 559 (2008).

12. H. J. Kang *et al.*, Spatio-temporal transcriptome of the human brain. *Nature* **478**, 483-489 (2011).

13. S. Liu *et al.*, Annotation and cluster analysis of spatiotemporal- and sex-related lncRNA expression in Rhesus macaque brain. *Genome Res*, (2017).

14. T. Negishi, Y. Ishii, S. Kyuwa, Y. Kuroda, Y. Yoshikawa, Primary culture of cortical neurons, type-1 astrocytes, and microglial cells from cynomolgus monkey (Macaca fascicularis) fetuses. *Journal of neuroscience methods* **131**, 133-140 (2003).

15. J. Li *et al.*, EZH2-mediated H3K27 trimethylation mediates neurodegeneration in ataxia-telangiectasia. *Nature neuroscience* **16**, 1745-1753 (2013).

16. M. J. de Hoon, S. Imoto, J. Nolan, S. Miyano, Open source clustering software. *Bioinformatics* **20**, 1453-1454 (2004).
